# The FBH family of bHLH transcription factors controls ACC synthase expression in sugarcane

**DOI:** 10.1093/jxb/ery083

**Published:** 2018-03-03

**Authors:** Valter Miotto Alessio, Natale Cavaçana, Luíza Lane de Barros Dantas, Nayoung Lee, Carlos Takeshi Hotta, Takato Imaizumi, Marcelo Menossi

**Affiliations:** 1 Departamento de Genética, Evolução, Microbiologia e Imunologia, Instituto de Biologia, Universidade Estadual de Campinas, CP, Campinas, SP, Brazil; 2 Departamento de Bioquímica, Instituto de Química, Universidade de São Paulo, São Paulo, SP, Brazil; 3 Department of Biology, University of Washington, Seattle, WA, USA

**Keywords:** ACC synthase, bHLH, ethylene biosynthesis, FBH, sucrose, sugarcane maturation, transcriptional regulation

## Abstract

Ethylene is a phytohormone involved in the regulation of several aspects of plant development and in responses to biotic and abiotic stress. The effects of exogenous application of ethylene to sugarcane plants are well characterized as growth inhibition of immature internodes and stimulation of sucrose accumulation. However, the molecular network underlying the control of ethylene biosynthesis in sugarcane remains largely unknown. The chemical reaction catalyzed by 1-aminocyclopropane-1-carboxylic acid synthase (ACS) is an important rate-limiting step that regulates ethylene production in plants. In this work, using a yeast one-hybrid approach, we identified three basic helix-loop-helix (bHLH) transcription factors, homologs of Arabidopsis FBH (FLOWERING BHLH), that bind to the promoter of *ScACS2* (Sugarcane *ACS2*), a sugarcane type 3 ACS isozyme gene. Protein–protein interaction assays showed that sugarcane FBH1 (ScFBH1), ScFBH2, and ScFBH3 form homo- and heterodimers in the nucleus. Gene expression analysis revealed that *ScFBHs* and *ScACS2* transcripts are more abundant in maturing internodes during afternoon and night. In addition, Arabidopsis functional analysis demonstrated that FBH controls ethylene production by regulating transcript levels of *ACS7*, a homolog of *ScACS2*. These results indicate that ScFBHs transcriptionally regulate ethylene biosynthesis in maturing internodes of sugarcane.

## Teaser

Identification of transcription factors that control the expression of the sugarcane *ACS* gene, which is likely involved in ethylene-controlled sucrose accumulation and the circadian regulation of ethylene biosynthesis.

## Introduction

Sugarcane (*Saccharum* spp.) is a monocot that accumulates large quantities of sucrose in the culm, reaching concentrations of up to 650 mM (Welbaum and Meinzer, 1990) or 18% of fresh weight (Inman-Bamber *et al.*, 2011). Sugarcane culm maturation is a continuous process of sucrose accumulation that forms a concentration gradient from the younger and immature internodes at the top to the older and mature internodes at the base (Moore, 1995; Rae *et al.*, 2005). Exogenous application of the plant hormone ethylene has a dramatic effect on sugarcane maturation by inducing sucrose accumulation in young internodes and inhibiting their growth (Chong *et al.*, 2010; Cunha *et al.*, 2017). Transcriptomic analysis has also identified ethylene as a potential regulator of sugar storage in the internodes (Papini-Terzi *et al.*, 2009; Cunha *et al.*, 2017). Since sucrose is the main product of sugarcane, understanding the molecular network involved in ethylene biosynthesis that affects sucrose accumulation should be very informative.

Ethylene synthesis starts with the formation of *S*-adenosylmethionine (SAM) from methionine through the action of SAM synthase. SAM is the substrate of ACC synthase (ACS) in the formation of 1-aminocyclopropane-1-carboxylic acid (ACC), which is converted into ethylene by ACC oxidase (ACO) (Yang and Hoffman, 1984). The reactions catalyzed by ACS and ACO are the two rate-limiting steps of ethylene synthesis (Yang and Hoffman, 1984; Alexander and Grierson, 2002; Van de Poel and Van Der Straeten, 2014). ACS is encoded by a multigene family in all plants investigated (Harpaz-Saad *et al.*, 2012). Arabidopsis has eight functional (ACS2, 4–9, and 11) and one non-functional (ACS1) ACS enzymes, which can form 25 different combinations of functional homo- and heterodimers (Tsuchisaka *et al.*, 2009). The ACS enzymes share high similarities in their catalytic domains and are classified into three groups based on the presence of regulatory features in their non-catalytic C-terminal regions. The type 1 ACS has a calcium-dependent protein kinase (CDPK) and three mitogen-activated protein kinase (MAPK) phosphorylation sites; the type 2 possesses only a single CDPK phosphorylation site; and the type 3 does not have any predicted phosphorylation sites (Harpaz-Saad *et al.*, 2012; Booker and DeLong, 2015). ACS is a cytosolic enzyme with a short half-life (Bleecker and Kende, 2000), and protein phosphorylation plays an important role in regulating its stability and degradation (Chae and Kieber, 2005; Lyzenga and Stone, 2012). Despite lacking any predicted phosphorylation sites on their C-terminal regions, the type 3 ACS proteins are destabilized by the ubiquitin ligase XB3 ORTHOLOG2 (XBAT32) in Arabidopsis (Lyzenga *et al.*, 2012), indicating that post-translational regulation of type 3 ACS function may also be important, as observed for type 1 and 2 ACS (Booker and DeLong, 2015).

Transcriptional and post-translational regulation of ACS is a key mechanism in controlling ethylene levels. Different ACS members have unique and overlapping spatiotemporal expression patterns, which are modulated by hormones, developmental stages, and biotic and abiotic stresses (Tsuchisaka and Theologis, 2004; Harpaz-Saad *et al.*, 2012). Brassinosteroids and cytokinin promote ethylene synthesis by inducing *ACS* expression and regulating protein stability (Yi *et al.*, 1999; Hansen *et al.*, 2009). Auxin induces the expression of seven functional *ACS* genes (*ACS2*, *4–8*, and *11*) and affects their spatial expression patterns in Arabidopsis (Yamagami *et al.*, 2003; Tsuchisaka and Theologis, 2004). Ethylene itself controls *ACS* and *ACO* expression during tomato fruit ripening (Barry *et al.*, 2000). Stressful conditions, such as cold, heat, flooding, drought, high salinity, wounding, and pathogen attack, also alter the transcription of distinct *ACS* genes in different plant species (Bleecker and Kende, 2000; Harpaz-Saad *et al.*, 2012). Wounding induces *ACS2*, *4*, *6*, *7*, and *8*, and inhibits *ACS1* and *ACS5* expression in Arabidopsis. Heat increases *ACS4* expression and alters the spatial expression patterns of *ACS8* and *ACS11* (Tsuchisaka and Theologis, 2004). Hypoxia induces ethylene production in maize roots through *ZmACS2* and *ZmACS7* activation (Gallie *et al.*, 2009).

Although the developmental and environmental cues that lead to changes in ethylene production are well described, the list of transcriptional regulators that function downstream of these stimuli is still somewhat limited. For instance, the MADS-box transcription factors MaMADS5 and RIN regulate *MaACS1* and *LeACS2* during fruit ripening in banana and tomato, respectively (Ito *et al.*, 2008; Martel *et al.*, 2011; Roy Choudhury *et al.*, 2012). The tomato and tobacco ethylene response factors LeERF2/TERF2 are positive regulators in the feedback loop of ethylene production (Zhang *et al.*, 2009). The Arabidopsis WRKY33 activates *ACS2* and *ACS6* expression in response to pathogen invasion (Li *et al.*, 2012).

Recently, Yin *et al.* (2015) reported PhFBH4, a member of subgroup 16 of the bHLH transcription factor family, as a regulator of *PhACS1* at flower senescence in petunia. Subgroup 16 of the bHLH family in Arabidopsis comprises FLOWERING BHLH 1 (FBH1), FBH2, FBH3, and FBH4, a group of transcription factors that are preferentially expressed in vascular tissues, bind to the E-box *cis*-element (CANNTG) to activate *CONSTANS* (*CO*) transcription, and consequently promote flowering. FBH1 was also implicated as a mediator of circadian clock responses to temperature changes by regulating *CIRCADIAN CLOCK ASSOCIATED 1* (*CCA1*) expression (Nagel *et al.*, 2014). Transcription factors controlling ethylene synthesis genes in monocots, including sugarcane, are still largely unknown.

Modern sugarcane cultivars are interspecific hybrids between *Saccharum officinarum* and *Saccharum spontaneum* with highly complex polyploid and aneuploid genomes (Grivet and Arruda, 2002; Souza *et al.*, 2011). In this context, functional genomics studies are challenging and therefore scarce. Although some transcriptomic and proteomic studies have been performed, the molecular networks controlling physiological processes in sugarcane, such as ethylene synthesis, are not well characterized. In this study, we aimed to identify transcription factors that control the expression of *ScACS2*, a sugarcane type 3 ACS isozyme. We identified three bHLH transcription factors (ScFBH1, ScFBH2, and ScFBH3), which are phylogenetically related to Arabidopsis FBHs, dimerize in cell nucleus, and bind to the *ScACS2* promoter, activating its expression. We also found that *ScACS2* and *ScFBHs* are more abundantly expressed in maturing internodes during the afternoon and night. Functional studies using Arabidopsis as a model support our hypothesis of which FBHs are transcriptional regulators of *ACS2* in sugarcane. Our results provide the molecular link between the circadian clock and internode maturation through the regulation of *ScACS2* in sugarcane.

## Materials and methods

### Phylogenetic analysis

To investigate the phylogenetic relationship of sugarcane ScACS2 (NCBI accession ADZ96244.1), we retrieved ACS-related protein sequences from sorghum (*Sorghum bicolor*), maize (*Zea mays*), foxtail millet (*Setaria italica*), and switchgrass (*Panicum virgatum*) databases available on Phytozome (www.phytozome.net) and performed the alignment using the WebPRANK global alignment tool (Löytynoja and Goldman, 2010) with default parameters. Arabidopsis and tomato ACS were included to guide the classification of subgroups (Supplementary Table S1 at *JXB* online). The phylogenetic tree was constructed in MEGA6 (Tamura *et al.*, 2013) software using the neighbor-joining method with 1000 bootstrap replicates. Orthologous groups were deduced by a bidirectional best hit approach. To classify sugarcane FBH proteins, the bHLH domains of Arabidopsis FBH1 (At1g35460), FBH2 (At4g09180), FBH3 (At1g51140), FBH4 (At2g42280), AtbHLH128 (At1g05805), AtbHLH129 (At2g43140), AMS (At2g16910), GL3 (At5g41315), ICE1 (At3g26744), PIF3 (At1g09530), PIF4 (At2g43010), SPT (At4g36930), and TT8 (At4g09820), and sorghum Sb02g028300, Sb03g04286, and Sb07g027810 proteins were aligned, and the phylogenetic tree was constructed as described.

### Promoter isolation and validation

To isolate the *ScACS2* gene promoter (accession MF383332) without a sugarcane reference genome, we aligned the 1.5 kb promoter region of sorghum and maize *ScACS2* putative orthologous genes (locus ID Sb03g03070 and GRMZM2G163015, respectively) and identified conserved regions to design a forward primer for PCR amplification. The reverse primer was designed inside the *ScACS2* coding sequence. Primer sequences are listed in Supplementary Table S2. Genomic DNA was extracted from sugarcane RB855156 cultivar using the protocol described by Aljanabi *et al.* (1999) and used as a template for PCR amplification. The unique 1.3 kb PCR fragment was cloned into the pTZ57R/T (Thermo Fisher, USA) vector and sequenced. The transcriptional activity of the *ScACS2* promoter was assessed by a transient β-glucuronidase (GUS) histochemical assay. For this purpose, the promoter sequence was cloned into the pHGWFS7 vector (Karimi *et al.*, 2002) and transformed into *in vitro* grown sugarcane leaves and calli by particle bombardment following previously defined parameters (Gallo-Meagher and Irvine, 1993). Histochemical GUS staining was performed according to the method described by Jefferson *et al.* (1987). The samples were then transferred to ethanol 70% to remove chlorophyll.

### Promoter *in silico* analysis

To identify putative conserved regulatory motifs in the *ScACS2* promoter, its sequence was compared with sequences of orthologous promoters of *Sorghum bicolor* (Sb03g03070), *Zea mays* (GRMZM2G163015), *Setaria italica* (Si001369m), and *Panicum virgatum* (Pavirv00040519m). Promoter sequences were selected by retrieving a 1 kb region upstream of the translational start site using Phytozome genomic data and alignments were performed by using BlastN, with the recommended parameters described by Kaplinsky *et al.* (2002), LAGAN (Brudno *et al.*, 2003), and MULAN (Ovcharenko *et al.*, 2005) algorithms. Conserved promoter elements present in at least three different species, including sugarcane, were considered in the search for putative transcription factor binding sites (TFBS). We identified putative TFBS using the PlantPAN 2.0 database (Chow *et al.*, 2016), and the resulting *cis*-element sequences were manually double-checked against original references; elements containing inconsistencies were discarded.

### Yeast one-hybrid assay

Yeast one-hybrid (Y1H) assays were performed using the Matchmaker Gold Yeast One-Hybrid System (Clontech, USA). The 1 kb region of the *ScACS2* promoter (upstream from the translational start site) was cloned into the pAbAi vector and transformed into the yeast Y1HGold strain to generate the bait reporter strain. The minimal inhibitory concentration of aureobasidin A (AbA) and auto-activation tests were performed. To produce cDNA libraries, total RNA was extracted from leaf +1 (the highest unfolded leaf with a visible dewlap) and culm of sugarcane plants (cultivar RB855156) using an RNeasy Plant Mini kit (Qiagen, USA). Each cDNA library was prepared with a pool of RNA samples from five different plants according to the manufacturer’s instructions (Clontech, USA). Both libraries were screened, and after three rounds of re-streaking each colony, the positive clones were sequenced and the putative function of the proteins was determined by BLAST searches on the NCBI database.

### Y1H confirmation

Complete coding sequences of *ScFBH1* (accession MF383333), *ScFBH2* (accession MF383334), and *ScFBH3* (accession MF383335) were cloned into the *Nde*I and *Eco*RI sites of the pGADT7 vector fused in-frame with the Gal4 activation domain (AD). The ScFBH1 and ScFBH3 coding sequences were codon-optimized for yeast (*Saccharomyces cerevisiae*) usage (accessions MF383336 and MF383337). Plasmids were transformed into the yeast strain containing the *ScACS2* promoter by the lithium acetate/PEG-mediated method (Gietz and Woods, 2006). Transformed yeast bait cells were plated on to SD/-Leu and SD/-Leu/AbA^200^ media and incubated for 5 days at 30 °C.

### bHLH domain three-dimensional modeling

The three-dimensional homology models of the bHLH domain of Arabidopsis and sugarcane FBHs were generated with the SWISS-MODEL workspace (www.swissmodel.expasy.org; Biasini *et al.*, 2014), based on sequence alignment with the 4ATK structure deposited on Protein Data Bank (www.rcsb.org). The quality of the homology models was assessed by QMEAN6 Z-scores (0< |Z-score| <1) and a Ramachandran plot considering more than 90% of residues in the most favored regions. The models were superimposed and visualized using Chimera 1.11.2 software (www.rbvi.ucsf.edu/chimera; Pettersen *et al.*, 2004).

### Electrophoretic mobility shift assay

A recombinant glutathione *S*-transferase (GST)-fused ScFBH2 protein was generated using the full-length *ScFBH2* coding sequence cloned into the pGEX4T-1 vector (GE Healthcare Life Sciences, USA). To induce the expression of the recombinant GST and GST-ScFBH2 proteins, 0.5 mM of isopropyl β-D-1-thiogalactopyranoside was added to each bacterial culture (at OD_600_=~0.3). After incubation for 20 h at 16 °C, the cell culture was collected by centrifugation and resuspended in buffer containing 20 mM HEPES·KOH, pH 7.2, 80 mM KCl, 0.1 mM EDTA, 0.1 mM PMSF, 1 mM DTT, and Pierce Phospatase Inhibitor Mini Tablets (Thermo Scientific, USA). After sonication, the whole extracts were collected by centrifugation and used for electrophoretic shift mobility assay (EMSA).

A total of 4 μg of cell extracts containing GST or GST-ScFBH2 proteins was incubated with 50 nM of a Cy5-labeled *ScACS2* promoter probe in binding buffer (20 mM HEPES·KOH, pH 7.2, 80 mM KCl, 0.1 mM EDTA, 5% (v/v) glycerol, 2.5 mM DTT) with 0.2 μg/μl BSA, and 500 ng poly(dI·dC). For the competition, a 5-, 10-, or 20-fold molar excess of unlabeled competitor DNA to the Cy5-labeled probes was added. After incubation for 30 min at room temperature, samples were separated by electrophoresis on a 7% native polyacrylamide gel in 0.25×TBE. Fluorescent gel images were obtained by using a Typhoon FLA 9000 Biomolecular Imager (GE Healthcare Life Sciences, USA). The probe sequence of the *ScACS2* promoter is 5′-[Cy5] CCTGCTTGCA*CACTTG*CAC*CACTTG*TGCGTGAGCC-3′ (two E-boxes are shown in italics), and the competitor sequence with mutation in two E-boxes is 5′-CCTGCTTGCA**A CCTCA**CAC**ACCTCA**TGCGTGAGCC-3′ (mutated E-boxes are shown in bold).

### Yeast two-hybrid assay

Yeast two-hybrid (Y2H) assays were performed using the Matchmaker Gold Yeast Two-Hybrid System (Clontech, USA). The coding regions of *ScFBH1*, *ScFBH2*, and *ScFBH3* were cloned into pGADT7 and pGBKT7 for in-frame expression with the Gal4 AD and DNA-binding domain (BD), creating all combinations of baits and preys (primers are shown in Supplementary Table S2). Cloned genes had no transcriptional activation activity in yeast cells (data not shown). Bait and prey construct pairs were then co-transformed into the yeast strain Y2H-Gold (Gietz and Woods, 2006). Yeast cells were grown on double dropout medium (DDO: SD/-Leu/-Trp) and quadruple dropout (QDO: SD/-Leu/-Trp/-His/-Ade) for 5 days at 30 °C. We tested all nine possible interactions between the three ScFBHs.

### Subcellular localization and bimolecular fluorescence complementation

*ScFBH1*, *ScFBH2*, and *ScFBH3*, previously cloned into the pENTR/D-TOPO or pDONR221 entry vectors (Thermo Fisher, USA), were cloned into the pSITE-3CA vector for analyzing their subcellular localization, and into the pSITE-cEYFP-C1 and pSITE-nEYFP-C1 vectors for bimolecular fluorescence complementation (BiFC) (Martin *et al.*, 2009). The construct RFP:NbH2B (Chakrabarty *et al.*, 2007) was used as a transformation control and nuclear localization reference. The plasmids were introduced into *Agrobacterium tumefaciens* strain GV3101 and after being grown overnight at 28 °C, cultures were suspended in infiltration buffer (10 mM MES, pH 5.6, 10 mM MgCl_2_, and 200 µM acetosyringone) to an OD_600_ of 0.5. After 3 h incubation, the *Agrobacterium* suspensions were injected into *Nicotiana benthamiana* leaves. Three days after infiltration, yellow fluorescent protein (YFP) and red fluorescent protein (RFP) fluorescence was detected by a Leica SP5 confocal microscope using a previously described method (Martin *et al.*, 2009). For BiFC analysis, we tested all possible combinations between the three ScFBHs, and we tested their interaction with GST protein as a negative control.

### Transient luciferase reporter assay

The *ScACS2* promoter (1 kb) was cloned into the pFLASH vector (Kubota *et al.*, 2017) to generate a luciferase reporter plasmid. The *ScFBHs* constructs in the pSITE-3CA vector described above were used as effectors. The 35S promoter-driven Renilla luciferase plasmid was used as a control to measure the efficiency of transient transfection (Fenske *et al.*, 2015). Transient transfection of *N. benthamiana* was performed as described above. Total soluble proteins were extracted from leaf discs and luciferase activities were analyzed using the Dual-Luciferase Reporter Assay System (Promega, USA). The luminescence was measured using a SpectraMax M3 plate reader. Data were presented as the means ±SE obtained from three biological replicates. Significant differences of means were assessed using Tukey’s test (*P*<0.05).

### RNA extraction and quantitative RT-PCR

To quantify mRNA levels of the sugarcane *ScACS2* and *ScFBHs* genes, the leaf +1, internode 1 (immature), and internode 5 (maturing) were harvested from 9-month-old field-grown sugarcane plants (cultivar SP803280) in summer 2013. Three biological replicates of each organ were harvested at 2 h intervals, starting 30 min after dawn (5.30 am) for a total of 12 time points during the day. The plant material was ground in liquid nitrogen and 100 mg of tissue was used for total RNA preparation using Trizol (Life Technologies, USA). RNA samples were treated with DNase I (Life Technologies, USA) and purified with the RNeasy Plant Mini Kit (Qiagen, USA). The cDNA was synthesized from 5 μg of total RNA using the SuperScript III First Strand Synthesis System (Life Technologies, USA) and diluted 10-fold. Quantitative PCRs (qPCRs) were performed using the Fast SYBR Green PCR Master Mix (Life Technologies, USA) on a Fast 7500/7500 Real-Time PCR System (Life Technologies, USA). Each reaction contained 6 μl of Fast SYBR Green Master Mix, 3 μl of H_2_O, 2.4 μl of each gene-specific primer pair (10 μM), and 0.6 μl of 10-fold diluted cDNA. We included negative controls (water) to confirm the absence of any contaminant. The specificity and efficiency of the *ScFBH1*, *ScFBH2*, *ScFBH3*, and *ScACS2* primers (see Supplementary Table S2) were assessed by constructing serial dilution standard curves and by melting curve analysis. The sugarcane *GAPDH* gene was used as a reference (Iskandar *et al.*, 2004). Each data value presented is the ΔCt (Livak and Schmittgen, 2001) mean ±SE derived from three biological replicates, and the value of each replicate is the mean of two technically replicated qPCR reactions.

For the Arabidopsis experiment, plants overexpressing the Arabidopsis homologs *FBH1* (*35S:AtFBH1* #24–53) and *FBH4* (*35S:FBH4* #21–6), as well as the *fbh* quadruple mutant (*35S:amiRFBH1-2*, *fbh2-1*, *fbh3-1*, and *35S:amiRFBH4*-3 #2–5), were previously obtained and characterized (Ito *et al.*, 2012; Kinmonth-Schultz *et al.*, 2016). Seedlings were grown on plates containing 1×Linsmaier and Skoog (LS; Caisson, USA) medium, pH 5.8, and 1% sucrose (w/v) under long-day (LD) conditions (16 h light/8 h dark, at 22 °C) for 2 weeks, and harvested at 3 h intervals from 1 h to 22 h after the onset of light. To quantify mRNA levels of the Arabidopsis *ACS2*, *ACS4*, *ACS6*, *ACS7*, and *ACS8* genes, total RNA was isolated from seedlings using an illustra RNAspin Mini kit (GE Healthcare, USA) and treated with a Turbo DNA-free kit (Thermo Fisher, USA) to eliminate remnant DNA in the samples. To synthesize cDNA, 2 μg of total RNA was reverse-transcribed using an iScript cDNA synthesis kit (Bio-Rad, USA). cDNA was diluted 3-fold and 2 μl samples were used for qPCRs. The reactions were performed using a Bio-Rad MyiQ real-time detection system. Buffer composition details can be found in Ito *et al.* (2012). Negative controls (water) were included. The *ACS* primer sequences used for qPCR were previously described (Li *et al.*, 2012). Serine/threonine-protein phosphatase (PP2A) was used as an internal control for normalization (Hong *et al.*, 2010). Each value presented was calculated as described above for the sugarcane experiment.

### Arabidopsis hypocotyl and root length measurement

All hypocotyl and root measurement assays were performed with seedlings (Col-0, *fbh* quadruple mutant *#2–5*, *35S:FBH1 #24–53*, and *35S:FBH4 #21–6*) grown on 0.5× LS (Caisson, USA) medium containing 1% sucrose (w/v), 0.8% agar (Fisher, USA), pH 5.8, with 10 µM ACC (PhytoTechnology, USA) or 100 µM AgNO_3_ (Sigma, USA). The plates were chilled at 4 °C in the dark for 3 days to synchronize germination and then moved to 22 °C under LD conditions for 10 days and DD (continuous dark) for 4 days in a vertical position. Light was provided by full-spectrum white fluorescent bulbs with a fluence rate of 60–90 μmol m^–2^ s^–1^. Hypocotyl and root length was measured using ImageJ software (Schneider *et al.*, 2012). The data were analyzed by ANOVA followed by comparison of mean values using Tukey’s test. Data are presented as mean ±SD.

### Ethylene measurements

For ethylene measurements, four Arabidopsis seedlings of each genotype (Col-0, *fbh* quadruple mutant *#2–5*, *35S:FBH1 #24–53*, and *35S:FBH4 #21–6*) were grown in LD conditions on 0.5× Murashige and Skoog (Sigma, USA) medium containing 1% sucrose (w/v) and 0.8% agar (Fisher, USA), pH 5.8, inside flasks capped with rubber serum stoppers. After 2 weeks, flasks were opened for 1 h and then closed to allow ethylene accumulation. After 24 h, 1 ml of head space was withdrawn and analyzed using a gas chromatograph equipped with a flame ionization detector (HP-6890) as described by Arraes *et al.* (2015). Each value reported represents the average of four biological replicates ±SD. The data were analyzed by ANOVA followed by comparison of mean values using Tukey’s test. Data are presented as mean ±SD. The total ethylene production was expressed as pl h^–1^.

## Results

### The *ScACS2* promoter is transcriptionally active and contains multiple transcription factor binding sites

As a first step to identify the transcription factors able to bind to the *ScACS2* promoter, we confirmed the transcriptional activity of the promoter by transient transformation of a *promScACS2:GUS* construct into *in vitro*-grown sugarcane leaves and calli by particle bombardment. Histochemical staining showed several GUS foci on sugarcane leaves and calli transformed with the *PUBI:GUS* (positive control) and *promScACS2:GUS* constructs (Supplementary Fig. S1), confirming the transcriptional activity of the isolated *ScACS2* promoter sequence. No GUS foci were observed on negative controls.

The ACS phylogenetic analysis revealed that ScACS2 clustered with Arabidopsis ACS7 and other putative type 3 ACSs from different species (Supplementary Fig. S2). This result is consistent with previous ACS phylogenies (Gallie and Young, 2004; Booker and DeLong, 2015). To identify conserved *cis*-elements present in the *ScACS2* promoter, we compared its sequence with the promoter sequences of orthologous *ACS* genes from sorghum, maize, foxtail millet, and switchgrass identified by the phylogenetic analysis. Several conserved regions were found, suggesting the presence of common regulatory *cis*-elements on type 3 *ACS* gene promoters among different grasses (Supplementary Fig. S3). Within these conserved promoter sequences, we found putative TFBS functionally involved in physiological processes associated with ethylene. These TFBS are involved with plant development (root hair and endosperm-specific expression elements), hormone signaling (BZR1-binding site), response to light (GATA and SORLIP elements), and response to stresses such as dehydration (MYB2 binding site), hypoxia (ANAERO1 element), and pathogen attack (WRKY biding sites). We also identified putative bHLH transcription factors binding sites (E-boxes) and ARF and Golden2-like binding sites (Supplementary Table S3).

As we identified a transcriptionally active promoter sequence of the *ScACS2* gene, we next aimed to identify potential transcription factors that bind to the sequence using Y1H screening. We prepared two Gal4 AD fused cDNA libraries derived from sugarcane leaf and culm to screen proteins that can bind to the 1 kb region of the *ScACS2* promoter. Approximately 360,000 clones were screened and 11 different clones retained their ability to grow in selective media (-Leu/+Aba^200^) after being re-streaked for three generations (see Supplementary Table S4 for proteins identified in the screening). Not surprisingly, the Y1H screening identified proteins able to bind to the *ScACS2* promoter, including proteins similar to ARF, Golden2-like, and three different bHLH domain-containing transcription factors. As these bHLHs shared high levels of similarity with Arabidopsis bHLHs named as FBHs, we named the three different sugarcane bHLH transcription factors ScFBH1, ScFBH2, and ScFBH3.

### ScFBH proteins bind to the *ScACS2* promoter and localize in the cell nucleus

**Fig. 1.**
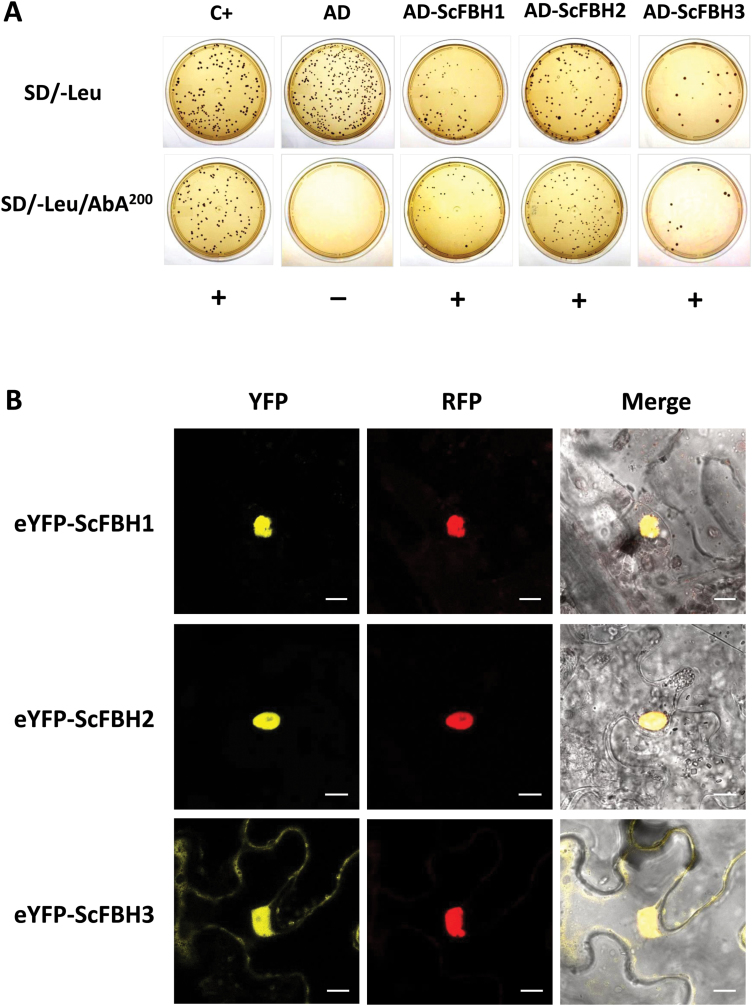
ScFBH1, ScFBH2, and ScFBH3 bind to the *ScACS2* promoter and localize in the nucleus. (A) Protein–DNA interaction between full-length ScFBH1, ScFBH2, and ScFBH3 proteins and the *ScACS2* promoter. The yeast strain harboring the aureobasidin (AbA) resistance gene controlled by the *ScACS2* promoter was transformed with pGADT7 vector expressing ScFBH1, ScFBH2, and ScFBH3 fused to the Gal4 activation domain (AD). Transformants were selected by plating on SD/-Leu media. Positive interactions (indicated by +) were identified by the capacity of the yeast to grow on SD/-Leu media containing AbA. The positive control (C+) consists of the interaction of p53 with its recognition site. The negative control indicates that the AD domain alone is unable to bind to the *ScACS2* promoter (indicated by –). (B) Subcellular localization of ScFBH1, ScFBH2, and ScFBH3. Constructs containing the *ScFBH1*, *ScFBH2*, and *ScFBH3* genes fused to eYFP were transiently transformed into *N. benthamiana* epidermal leaf cells by *Agrobacterium* infiltration, and fluorescence was observed using a confocal microscope. The construct RFP:NbH2B (Chakrabarty *et al.* 2007) was co-transformed as a control of transformation efficiency and to mark the location of the nucleus. The merged images are a digital merge of bright-field and fluorescent images. Bars=10 μm. (This figure is available in color at *JXB* online.)

Based on the protein homologs from sorghum and Arabidopsis, we noticed that our bHLH domain-containing Y1H clones did not harbor the complete coding sequences. In addition, none of them had the corresponding physical clone with the complete sequence available in the sugarcane EST collection (SUCEST project, www.sucest-fun.org). In order to characterize the function of these bHLH proteins, we cloned the complete coding sequences of *ScFBH1*, *ScFBH2*, and *ScFBH3* based on the sequences available from the sugarcane genome project (SUCEST). To confirm whether the full-length bHLH proteins could bind to the *ScACS2* promoter, we performed Y1H assays using the full length of proteins. The complete coding DNA sequences of *ScFBH1*, *ScFBH2*, and *ScFBH3* were fused to the Gal4 AD and expressed in the yeast strain harboring the AbA resistance gene under the control of the *ScACS2* promoter. Expression of AD-ScFBHs activated the reporter gene, which promoted yeast growth on the selective medium containing aureobasidin (SD/-Leu/AbA) (Fig. 1A).

Next, we determined the subcellular localization of ScFBH proteins. Fluorescence signals from chimeric ScFBH proteins fused to YFP were analyzed by confocal microscopy in transiently transformed *N. benthamiana* epidermal cells (Fig. 1B). In all cases, strong YFP signals concentrated in the cell nucleus, as confirmed by the co-expression of *N. benthamiana* histone H2B fused to RFP. This result showed that ScFBH1, ScFBH2, and ScFBH3 were localized in the nucleus, supporting their role as transcription factors.

### ScFBHs are closely related to Arabidopsis FBHs

**Fig. 2.**
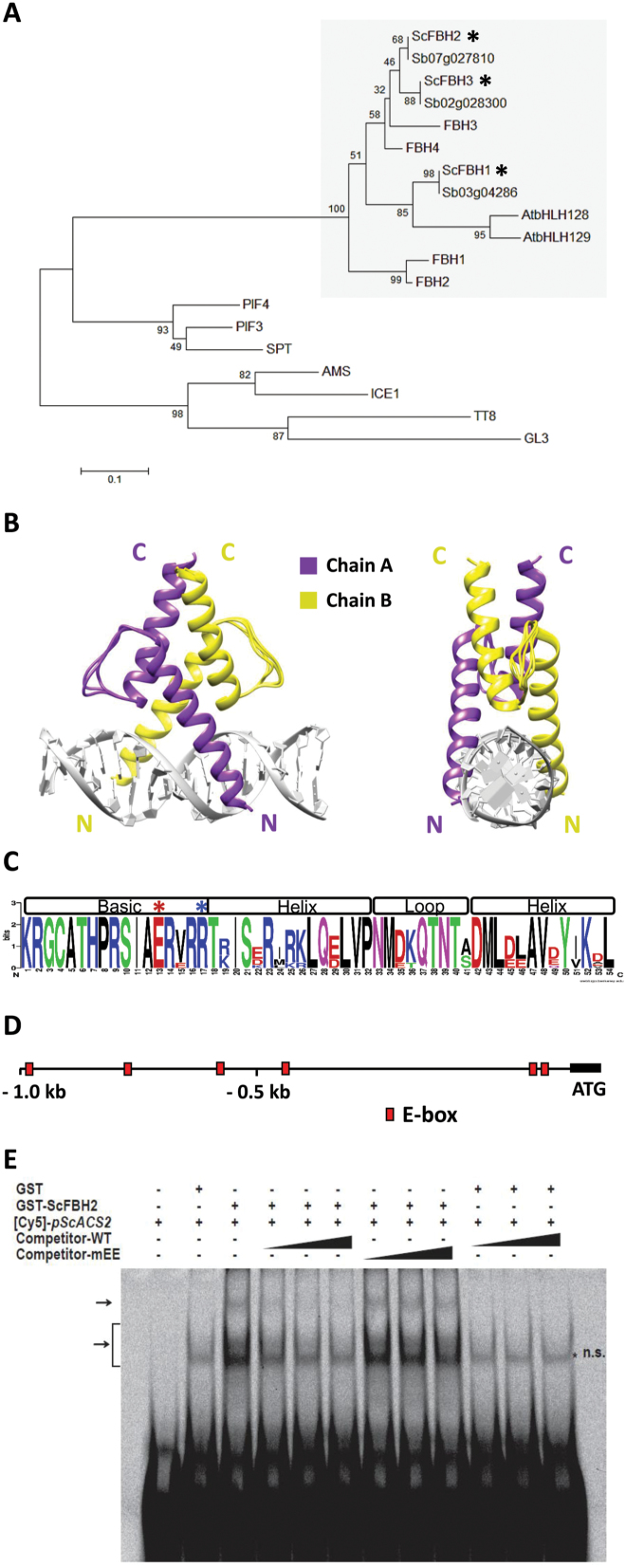
Sugarcane FBHs (ScFBHs) are phylogenetically related to Arabidopsis FBH, and have conserved sequences and structure features typical of the bHLH domain. (A) Phylogenetic tree of ScFBH1, ScFBH2, ScFBH3, and other known bHLH transcription factors (AMS, ABORTED MICROSPORES; FBH, FLOWERING BHLH; GL3, GLABRA 3; ICE 1, INDUCER OF CBF EXPRESSION 1; PIF, PHYTOCHROME INTERACTING FACTOR; SPT, SPATULA; TT8, TRANSPARENT TESTA 8). The shaded box represents bHLH subfamily 16 (Toledo-Ortiz *et al.*, 2003). Asterisks indicate the positions of ScFBH1, ScFBH2, and ScFBH3. (B) Structure superposition of the three-dimensional models of ScFBH1, ScFBH2, ScFBH3 and Arabidopsis FBH1, FBH2, FBH3, and FBH4, demonstrating the structural conservation of the bHLH DNA-binding domain. The quaternary structure was predicted by SWISSMODEL workbench. N and C indicate the N- and C-terminal regions, respectively. (C) Protein sequence logo (WebLogo, weblogo.berkeley.edu/logo.cgi) generated from the alignment of ScFBH and Arabidopsis FBH bHLH domains. The asterisks indicate the Glu (E)-13 and Arg (R)-17 residues involved in E-box interaction. (D) Distribution of E-box DNA motifs in the 1 kb region of the *ScACS2* gene promoter. The positions of E-box elements are indicated by the boxes. (E) Electrophoretic mobility shift assay showing the direct binding of GST-tagged ScFBH2 (GST-ScFBH2) protein to the *ScACS2* promoter. The 35 nt fragment containing two E-boxes in the *ScACS2* promoter was Cy5-labeled ([Cy5]-*pScACS2*). The increasing concentrations of unlabeled competitors (5×, 10×, and 20×) containing either wild-type (WT) or mutant (mEE) E-boxes are indicated by the triangles. GST was used as a negative control. Arrows indicate ScFBH2–DNA complexes. The asterisk indicates non-specific signal (n.s.). (This figure is available in color at *JXB* online.)

Our phylogenetic analysis revealed that ScFBHs belong to subfamily 16 of the bHLH transcription factor family (according to the classification of Toledo-Ortiz *et al.*, 2003), the same subfamily as the Arabidopsis FBHs (Fig. 2A). ScFBH1, ScFBH2, and ScFBH3 contain 361, 385, and 424 amino acids, respectively, and one bHLH domain on their C-terminal regions. Amino acid identities within the bHLH domains between sugarcane and Arabidopsis FBHs ranged from 72.5% to 96.1%. Superposition of three-dimensional models of the bHLH domains of sugarcane and Arabidopsis FBHs demonstrates structure and sequence conservation in the bHLH motifs involved in DNA binding (Fig. 2B), including Glu-13 and Arg-17 residues (Fig. 2C), which are crucial to the E-box binding (Fisher and Goding, 1992; Shimizu *et al.*, 1997). Ito *et al.* (2012) determined that Arabidopsis FBHs bind to E-box (CANNTG) elements present on the *CO* promoter. The 1 kb region of the *ScACS2* promoter contains six E-boxes (Fig. 2D). We confirmed the direct binding of ScFBH2 to the E-box elements present in the *ScACS2* promoter by EMSA (Fig. 2E). A mutated version of the E-box used as a competitor did not disturb the ability of ScFBH2 to bind to the native sequence, as expected. The high conservation of the DNA-binding domain between Arabidopsis FBHs and ScFBHs, as well as the EMSA results, provide clear evidences that ScFBHs interact with the *ScACS2* promoter by binding to E-box elements.

### ScFBHs form homo- and heterodimers to activate *ScACS2* expression

**Fig. 3.**
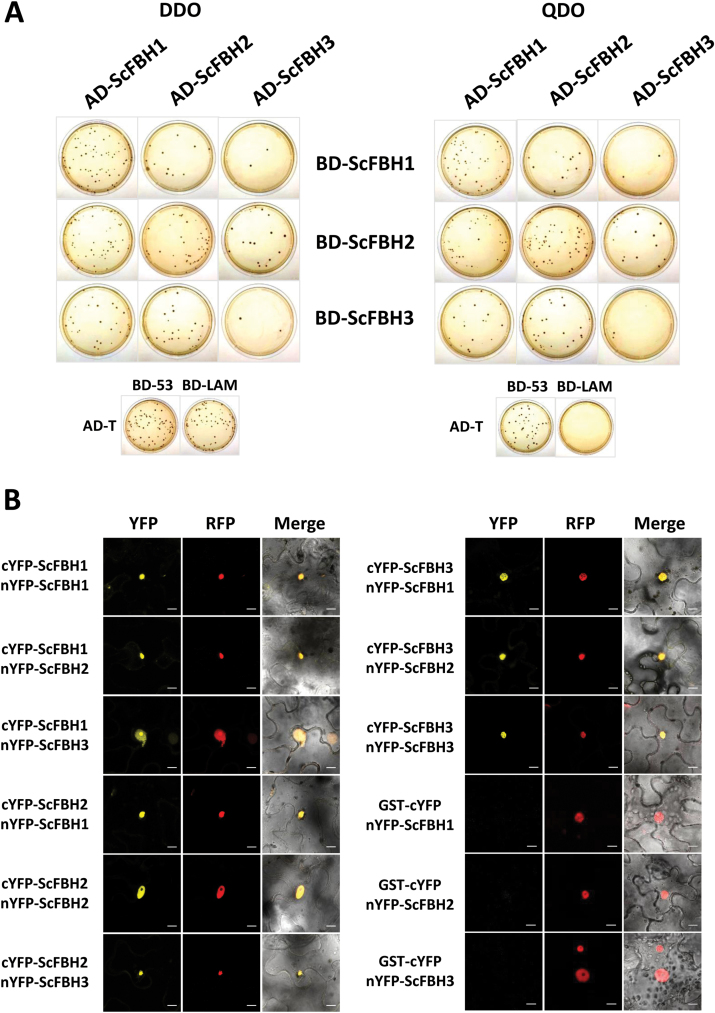
Homo- and heterodimerization of ScFBH1, ScFBH2, and ScFBH3. (A) Physical interaction between ScFBH1, ScFBH2, and ScFBH3 analyzed by Y2H assay. *ScFBH1*, *ScFBH2*, and *ScFBH3* were cloned into pGADT7 and pGBKT7 to generate AD and BD fusions. The Y2H-Gold yeast strain was co-transformed with all possible bait and prey combinations. The ability of yeast cells to grow on QDO (SD -Leu/-Trp/-His/-Ade) indicated positive interactions. Yeast cells transformed with AD-T + BD53 and AD-T + BD-LAM were included as positive and negative controls, respectively. (B) BiFC visualization of sugarcane bHLH interactions in tobacco leaf cells. ScFBH1, ScFBH2, and ScFBH3 were fused with the N- and C-termini of YFP and all nine possible interactions were tested. The interactions between ScFBHs fused with N-terminal YFP and GST fused with C-terminal YFP were tested as negative controls. The RFP:NbH2B construct (Chakrabarty *et al.* 2007) was co-transformed as a control of transformation efficiency and nuclear location. Bars=10 μm. (This figure is available in color at *JXB* online.)

The bHLH transcription factors usually function as homodimers or heterodimers (Heim *et al.*, 2003). Our three-dimensional homology modeling predicted the dimerization of ScFBHs (Fig. 2B). Therefore, we investigated the physical interaction between ScFBHs using the Y2H system. The *ScFBH* genes were cloned into the pGADT7 and pGBKT7 vectors to generate translational fusions with the Gal4 AD and BD. Auto-activation of any BD constructs was tested before conducting the Y2H assays, and it was not detected (data not shown). The AD and BD vector constructs were co-transformed into yeast; all nine possible combinations between the hybrid proteins were tested. Yeast grew in all tested interactions and in the positive control, while no growth was observed in the negative control (Fig. 3A). Few colonies grew in plates transformed with AD-ScFBH3 fusion; we attribute this to the cell toxicity presented by this protein in bacteria and yeast cells (data not shown).

In order to confirm the Y2H observations, we performed a BiFC assay. The genes *ScFBH1*, *ScFBH2*, and *ScFBH3* were cloned into pSITE-cEYFP-C1 (which contains a split eYFP C-terminal fragment) and pSITE-nEYFP-C1 (which contains a split eYFP N-terminal fragment) and transiently transformed into *N. benthamiana* epidermal cells by *Agrobacterium* infiltration. Again, in all nine possible combinations among the three tested chimeric proteins, we detected strong YFP fluorescence in the nucleus of transformed cells, where the nuclear reference RFP-NbH2B protein was also detected (Fig. 3B). No YFP signals were detected in cells co-expressing ScFBH1, ScFBH2, or ScFBH3 fused to the N-terminal fragment of YFP and GST fused to the C-terminal YFP fragment. The Y2H and BiFC results demonstrated that ScFBH1, ScFBH2, and ScFBH3 form all possible homo- and heterodimer combinations *in vivo* and that such interactions specifically occur in the nucleus.

**Fig. 4.**
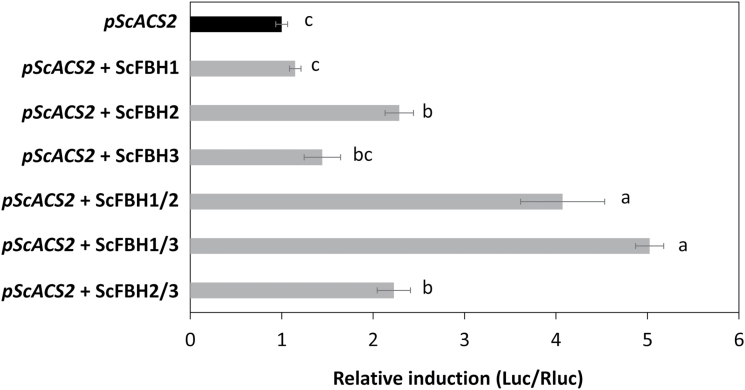
Luciferase reporter assay in *N. benthamiana*. The effects of ScFBH1, ScFBH2, and ScFBH3 homo- and heterodimers on firefly luciferase (Luc) activity controlled by 1 kb of the *ScACS2* gene promoter (*pScACS2*) were tested. The activity of Luc was normalized by the activity of Renilla luciferase (Rluc). Means were compared by ANOVA and Tukey’s test (*P*<0.05); the same letters to the right of the bars indicate that there was no statistically significant difference between treatments. Data represent means ±SE (n=4).

To investigate whether the binding of homo- and heterodimers of ScFBHs to the *ScACS2* promoter is important for the transcriptional regulation of *ScACS2*, we utilized the *ScACS2* promoter-controlled luciferase reporter system. When ScFBH1, ScFBH2, and ScFBH3 were transiently expressed in *N. benthamiana* epidermal cells, ScFBH2 significantly increased the activity of the luciferase reporter controlled by the *ScACS2* promoter (Fig. 4). All heterodimer combinations of ScFBHs significantly induced *ScACS2* promoter activity (Fig. 4). Together with the protein interaction assays, these results suggest that ScFBH proteins associate as dimers with the *ScACS2* promoter to induce the transcription of *ScACS2*.

### *ScFBHs* and *ScACS2* are expressed higher in maturing internodes

**Fig. 5.**
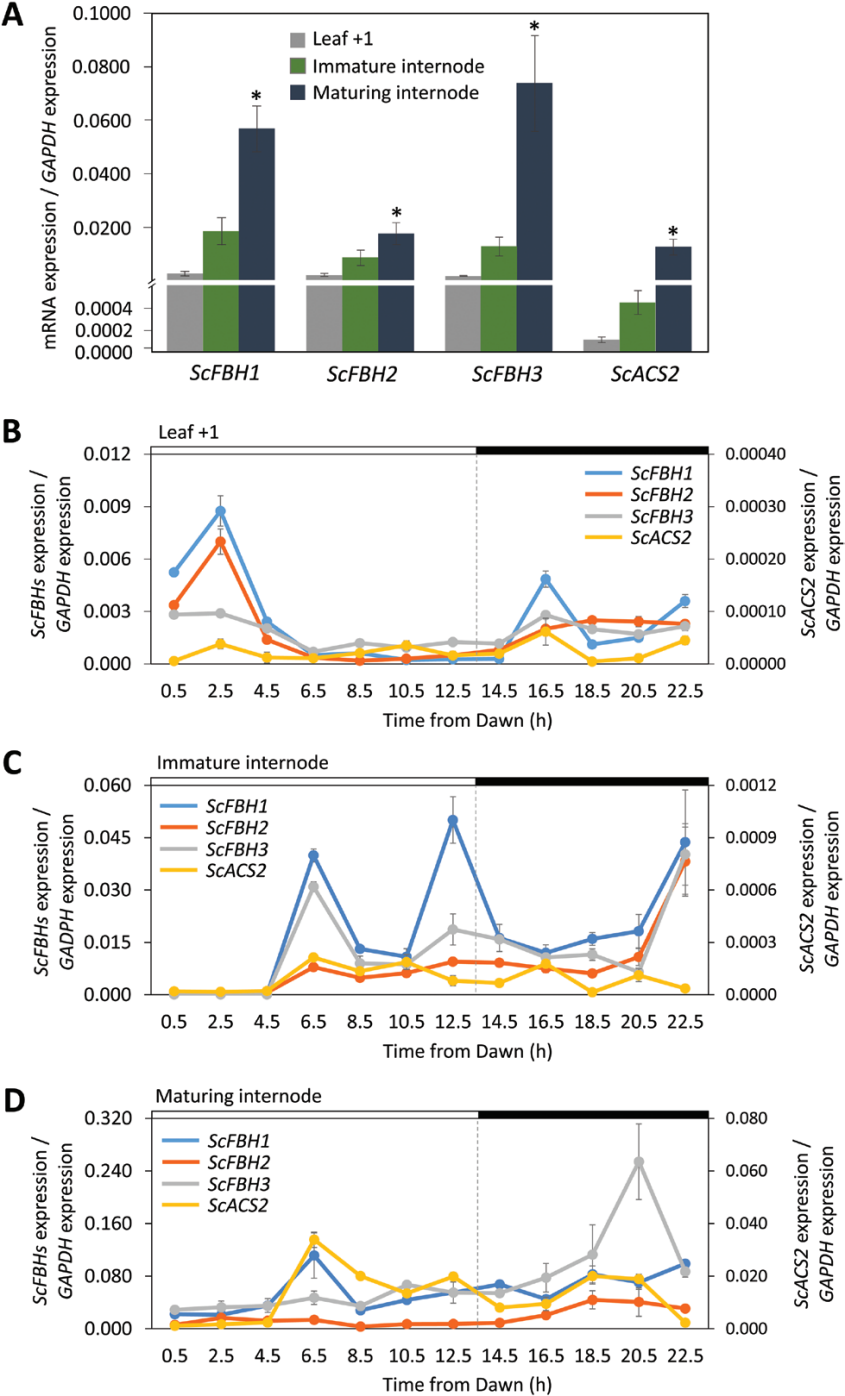
Expression profiles of *ScFBH1*, *ScFBH2*, *ScFBH3*, and *ScACS2* in different sugarcane organs and throughout the day. (A) Expression levels of *ScFBH1*, *ScFBH2*, *ScFBH3*, and *ScACS2* in leaf +1 and immature and maturing internodes. Bars represent the mean ±SE expression values from all time points. Asterisks indicate significant differences in mean gene expression between organs (*P*<0.05, Tukey’s test). (B–D) Daily expression profiles of *ScFBH1*, *ScFBH2*, *ScFBH3*, and *ScACS2* in leaf +1 (B), immature internode (C), and maturing internode (D). *ScACS2* expression values are presented on the secondary y-axis. Harvesting started 0.5 h after dawn (5.30 h) and was then repeated every 2 hours. The horizontal bars above the graphs represent periods of light (white bars) and darkness (black bars). Expression values were normalized using expression of the *GAPDH* gene (Iskandar *et al.*, 2004) as a reference, and the expression values were calculated by the ΔCt method (Livak and Schmittgen, 2001). Each point represents a mean ±SE (n=3). (This figure is available in color at *JXB* online.)

Next, we measured the mRNA levels of sugarcane *ScFBHs* and *ScACS2* in different plant organs throughout the day in order to analyze their spatiotemporal expression patterns. In sugarcane, the younger, immature internodes are located in the top part of the plant, whereas the oldest and mature internodes, with higher sucrose content, are close to the roots. Our qPCR analysis demonstrated that the expression patterns of both *ScFBHs* and *ScACS2* overlap in sugarcane organs. Low levels of *ScACS2* mRNA were detected in the leaf and in the immature internode, and the expression level was significantly higher in the maturing internode (Fig. 5A). We found similar results for *ScFBH1*, *ScFBH2*, and *ScFBH3*, that is, lower expression in the leaf and higher expression in the culm, reaching the highest levels in the maturing internode (Fig. 5A).

The overall daily expression patterns of *ScFBHs* were similar throughout the day (Fig. 5B–D). In the leaf, *ScFBH* expression levels peaked 2.5 hours after dawn, then reduced during the daytime, and increased again during the night (Fig. 5B). In the internodes, the expression levels of *ScFBHs* were low at dawn, and increased during the day (Fig. 5C and D). We observed the same pattern for *ScACS2* in maturing internode: its expression increased during the day, reaching the highest levels in the afternoon/evening (Fig. 5D).

### *FBH* overexpression in Arabidopsis induces typical ethylene-related phenotypes, increased *ACS7* expression, and ethylene production

**Fig. 6.**
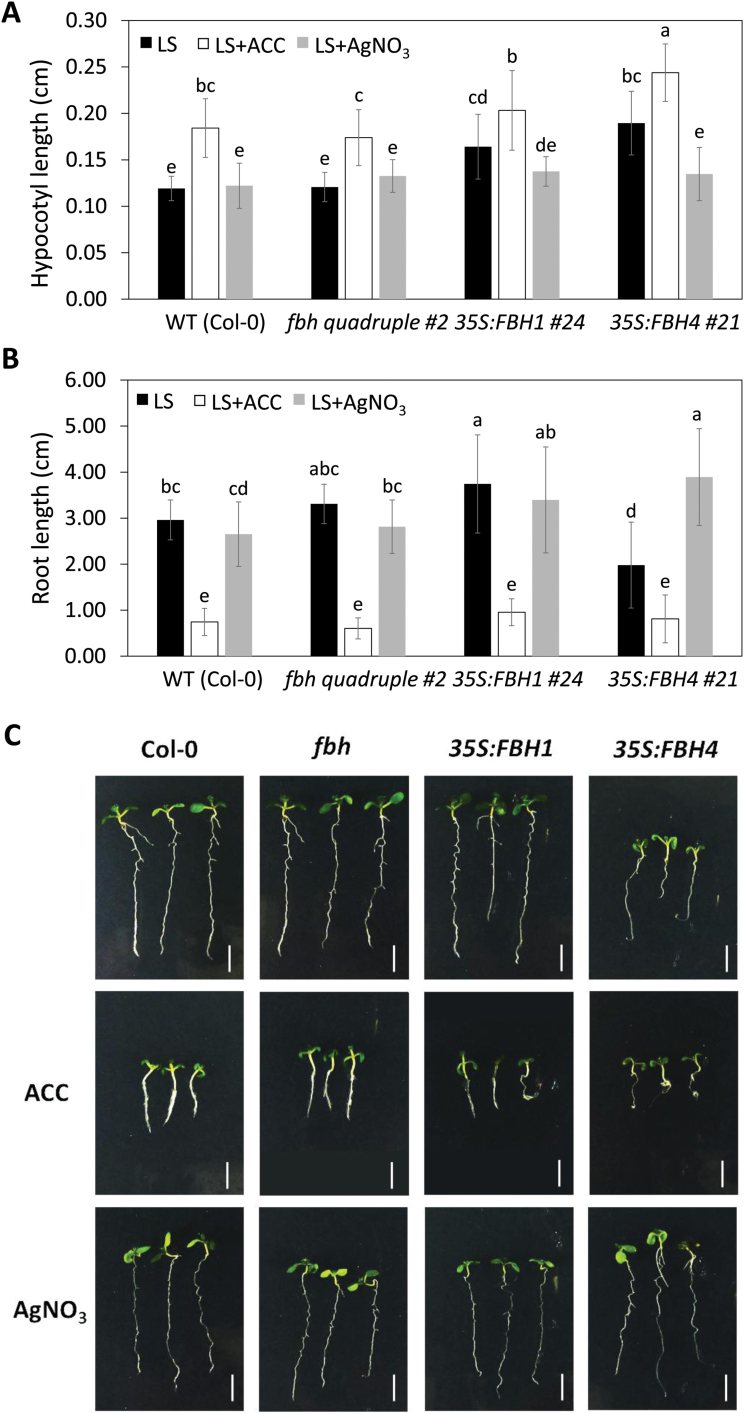
Effect of *FBH* overexpression and *fbh* loss of function on hypocotyl length, root length, and *ACS7* expression in Arabidopsis seedlings grown under long-day (LD) conditions. (A) Hypocotyl length and (B) root length of Col-0, *fbh* quadruple mutant, *35S:FBH1*, and *35S:FBH4* seedlings. After stratification, plants were grown on Linsmaier and Skoog media containing ACC (10 µM) or AgNO_3_ (100 µM) for 10 days at 22 °C under LD conditions. For each treatment, data presented are the means of 17–27 seedlings ±SD. Means were compared by ANOVA and Tukey’s test (*P*<0.05); the same letters above each bar indicate that there was no statistically significant difference between treatments. (C) Phenotypes of 10-day-old seedlings grown with and without ACC or AgNO_3_ under LD conditions. Bars=0.5 cm. (This figure is available in color at *JXB* online.)

As it is technically difficult to genetically study the function of genes in sugarcane, we decided to use Arabidopsis to assess whether FBH transcription factors regulate *ACS* gene transcription. We hypothesized that if Arabidopsis FBHs are also involved in *ACS* transcriptional regulation, overexpression and loss-of-function of *FBH* would change the expression levels of the *ACS* gene and potentially ethylene production. To assess this possibility, we first analyzed the hypocotyl and root length of *FBH1* and *FBH4* overexpressors and of the *fbh* quadruple mutant. Ethylene and ACC exposure can stimulate hypocotyl elongation (Smalle *et al*., 1997) and inhibit root growth (Ruzicka *et al*., 2007; Ivanchenko *et al.*, 2008) in light-grown seedlings. In dark-grown seedlings, ethylene inhibits hypocotyl and root elongation, exaggerates tightening of the apical hook, and induces swelling of the hypocotyl, in what is known as the triple-response phenotype (Guzmán and Ecker, 1990). Light-grown *35S:FBH1* and *35S:FBH4* seedlings exhibited significantly longer hypocotyls compared with wild-type seedlings (Fig. 6A), a phenotype that resembled the constitutive ethylene response mutant *ctr1* (Smalle *et al*., 1997), and *35S:FBH4* was defective in root growth (Fig. 6B and C). We also investigated the phenotypes of etiolated seedlings. Compared with wild-type seedlings, *fbh* mutant seedlings exhibited significantly longer hypocotyls, whereas *35S:FBH4* seedlings showed shorter and thicker hypocotyls (Supplementary Fig. S4). The hypocotyl phenotype was exaggerated when plants were grown in media containing ACC.

To determine whether the effects of *FBH* overexpression on Arabidopsis hypocotyl and root length were consequences of increased ACC production, we grew the plants on media containing AgNO_3_, an inhibitor of the ethylene response (Smalle *et al*., 1997; De Grauwe *et al.*, 2005). We found that AgNO_3_ treatment significantly decreased hypocotyl elongation in *35S:FBH1* and *35S:FBH4* plants when grown under the light regime (Fig. 6A). The AgNO_3_ treatment also blocked the root-shortening phenotype in *35S:FBH4* transgenic plants (Fig. 6B). These results support the notion that *FBH* overexpression promotes hypocotyl elongation and inhibits root growth by modulating ethylene production.

**Fig. 7.**
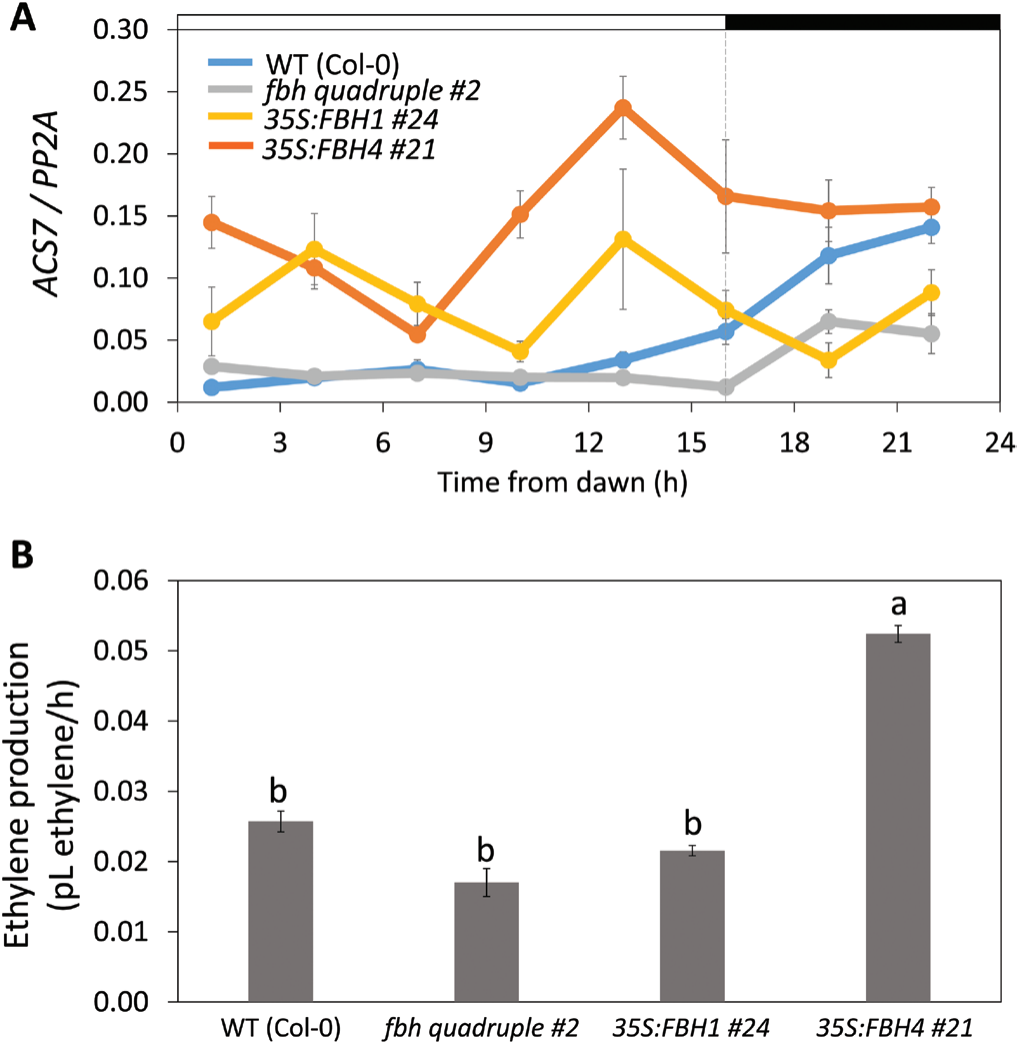
Effect of *FBH* overexpression and *fbh* loss of function on *ACS7* expression and ethylene production in Arabidopsis seedlings. (A) Expression of *ACS7* over the course of a day in wild-type (Col-0), *fbh* quadruple mutant, *35S:FBH1*, and *35S:FBH4* plants. The horizontal bars above the graphs represent periods of light (white bar) and darkness (black bar). The expression of the *PP2A* gene (Hong *et al.*, 2010) was used to normalize the data and the expression values were calculated by the ΔCt method (Livak and Schmittgen, 2001). Values represent means ±SE (n=3). (B) Ethylene production of wild-type, *fbh* loss-of-function mutant and FBH overexpressors. Data presented are the means ±SD of four replicates. Means were compared by ANOVA and Tukey’s test (*P*<0.05); the same letters above each bar indicate that there was no statistically significant difference between treatments. (This figure is available in color at *JXB* online.)

To elucidate the potential contribution of FBHs to *ACS* gene expression, we evaluated the daily expression pattern of *ACS2*, *4*, *6*, *7*, and *8* in *35S:FBH1* and *35S:FBH4* plants and the *fbh* quadruple mutant. As predicted, the expression levels of *ACS7* (the closest homolog of *ScACS2*) were elevated in the *35S:FBH* lines, especially during the subjective day, and reduced after 13 h from light onset in the *fbh* quadruple mutant compared with wild-type plants (Fig. 7A). We did not observe consistent changes in the expression patterns of *ACS2*, *4*, *5*, *6*, and 8 in transgenic plants compared with wild-type plants (Supplementary Fig. S5), except for a repression of *ACS8* in *fbh* quadruple mutant and *35S:FBH1* plants and induction of *ACS8* after 19 h in *35S:FBH4*, which resembled the observed effect on *ACS7* expression at night. Both *ACS7* and *ScACS2* are type 3 ACSs and exhibited a similar expression pattern throughout the day in Arabidopsis and sugarcane (Fig. 5D and 7A).

The analysis of ethylene production confirmed the differences observed in the phenotypic evaluation and *ACS* gene expression. Overexpression of *FBH4*, which produced the strongest ethylene-related phenotypes and induced higher levels of *ACS7*, also produced more amounts of ethylene (Fig. 7B).

## Discussion

Plant bHLH transcription factors comprise a large family, and are involved in the transcriptional control of various biological processes (Toledo-Ortiz *et al.*, 2003). Our Y1H screening identified three related bHLH transcription factors that bind to the *ScACS2* promoter (Fig. 1A and Supplementary Table S4) and act as transcriptional activators (Fig. 4). Phylogenetic analysis demonstrated that these proteins belong to subfamily 16 the of bHLH superfamily (Fig. 2A), to which four FBHs of Arabidopsis belong. Arabidopsis FBHs are transcriptional activators of *CO* and regulate photoperiodic flowering time. Ito *et al.* (2012) demonstrated that FBH1, FBH2, FBH3, and FBH4, by binding to the E-boxes, act in a redundant fashion on the *CO* promoter to activate its expression. FBH homologs identified in poplar and rice exhibited similar functions when overexpressed in Arabidopsis (Ito *et al.*, 2012; Song *et al.*, 2013). The amino acid alignment of the bHLH domains showed a high level of similarity between Arabidopsis FBHs and sugarcane ScFBHs. Both species’ FBHs possess conserved Glu-13 and Arg-17 residues (Fig. 2C), which are involved in the interaction with the E-box consensus CANNTG (Shimizu *et al.*, 1997; Toledo-Ortiz *et al.*, 2003). E-boxes exist on the *ScACS2* promoter (Fig. 2D) and are conserved among the promoters of homologous type 3 *ACS* genes from other grasses (Supplementary Table S3). EMSA analysis showed that ScFBH2 directly binds to E-boxes (CACTTG) present in the *ScACS2* promoter (Fig. 2E). Yin *et al.* (2015), meanwhile, found that the petunia homolog PhFBH4 is up-regulated during flower senescence and binds to the CACGTG motif of the *PhACS1* promoter to activate its expression. This finding is similar to our findings, indicating that the molecular link between FBHs and *ACS* might be conserved widely in both monocots and eudicots. Consistent with their role as transcription factors, all three ScFBH proteins are targeted to the nucleus (Fig. 1B).

The bHLH transcription factors are known to form homodimers and heterodimers, and to interact with members of other transcription factor families than the bHLH family to regulate the expression levels of target genes (Heim *et al.*, 2003; Toledo-Ortiz *et al.*, 2003). The three-dimensional modeling of ScFBHs and Arabidopsis FBHs predicted that they dimerize (Fig. 2B). We confirmed all nine possible combinations of interactions among sugarcane FBH proteins (ScFBH1, ScFBH2, and ScFBH3) by Y2H and BiFC assays (Fig. 3A and B). We also demonstrated that homo- and heterodimers activate the *ScACS2* promoter at distinct levels in the transient luciferase reporter assay (Fig. 4). This indicates that ScFBHs might bind as dimers on the E-boxes of the *ScACS2* promoter to activate its expression. Whether different combinations of ScFBH dimers act differently to regulate the transcription of target genes at different plant tissues and physiological conditions still needs to be investigated.

The gene expression analysis showed that *ScACS2* and *ScFBHs* transcript levels are low in leaves and immature internodes. However, *ScACS2* expression level is higher in the maturing internodes, where the expression levels of *ScFBHs* are also higher. Since the maturation process in sugarcane is characterized by a gradual increase in sucrose content from the apical to the basal internodes, our data revealed that the expression of *ScACS2* and *ScFBHs* correlates with plant development and the sucrose content of the internodes (Fig. 5A). Cunha *et al.* (2017) found that the application of ethylene to sugarcane plants induced sucrose synthase (SuSy) activity on maturing internodes a few days after the treatment. This ethylene treatment induced a 60% increase of sucrose levels approximately 1 month after the application, when the SuSy activity reduced to the level of activity in non-treated plants. The inhibition of ACS had the opposite effect, with treated plants exhibiting a 42% reduction in sucrose levels in the internodes compared with levels in untreated plants. In leaves, ethylene has no effect on SuSy activity or sucrose levels. In addition, in comparison with immature internodes, ETHYLENE-INSENSITIVE3-LIKE (EIL) homologs and one ACO homolog are repressed in mature internodes (Papini-Terzi *et al.*, 2009), indicating that there is a less active ethylene response in mature internodes; we believe that this represents a late stage of maturation. Interestingly, ethylene treatment induces *ScFBH1* in sugarcane internodes (Cunha *et al.*, 2017), which could indicate a feedback regulation of *ACS* by ethylene through the action of ScFBH1. Although the precise role of ethylene in sucrose accumulation in sugarcane needs to be studied further, our work supports the notion that ethylene acts as a modulator of the maturation process. Our findings imply that ScFBHs are a part of the network involved in the sucrose accumulation mediated by ethylene.

To determine the potential contribution of *ScFBHs* to the regulation of *ScACS2* throughout the day, we measured their daily expression patterns in leaves and immature and maturing internodes. In leaves, *ScFBHs* expression levels peak in the morning; however, the overall levels are very low relative to their expression levels in maturing internodes, and *ScACS2* expression remains constantly low throughout the day (Fig. 5B). In maturing internodes, where transcript levels of all genes are higher, the daily expression patterns of *ScACS2* and *ScFBHs* are similar. The overall expression pattern of *ScACS2* and *ScFBHs*, which is lower just after dawn and increases in the course of the day, resembles the expression pattern of *FBH4* in LD conditions (Ito *et al.*, 2012). The expression of FBH4 and possibly FBH1 has a diurnal oscillation pattern under constant light conditions, indicating the involvement of circadian regulation (Ito *et al.*, 2012). Circadian oscillation of ethylene synthesis has been described in Arabidopsis (Thain *et al.*, 2004), potato (Chincinska *et al.*, 2013), and sorghum (Finlayson *et al.*, 1999, 2004). In Arabidopsis, the *ACS6* promoter is the binding target of the core clock protein TOC1 (Huang *et al.*, 2012), and the rhythmic production of ethylene correlates with the expression pattern of *ACS8* (Thain *et al.*, 2004). Furthermore, FBH1 and the core clock protein CCA1 reciprocally regulate each other’s transcription (Nagel *et al.*, 2014). Taken together, these data suggest that ScFBHs may be involved in the circadian regulation of *ACS* expression in sugarcane and provide initial cues for further investigations.

In addition to the findings that ScFBHs bind to the *ScACS2* promoter in yeast and increase *ScACS2* promoter activity in *N. benthamiana* cells (Figs 1 and 4), the similar expression patterns of *ScFBHs* and *ScACS2* led us to hypothesize that ScFBHs act as direct transcriptional activators of *ScACS2*. We also hypothesize that this regulation may be conserved in other plants besides sugarcane, since FBHs can be found in various plant genomes (Ito *et al.*, 2012). We made use of Arabidopsis genetic resources to test this hypothesis. Ethylene and ACC production stimulate hypocotyl elongation (Smalle *et al*., 1997; Zhong *et al.*, 2012; Yu *et al.*, 2013) and inhibit root growth in light-grown Arabidopsis seedlings (Ruzicka *et al*., 2007; Negi *et al.*, 2008). We observed that overexpression of both *FBH1* and *FBH4* induced hypocotyl elongation in LD-grown Arabidopsis seedlings; this phenotype was reversed by the application of AgNO_3_, an ethylene antagonist (Fig. 6A). Additionally, etiolated *35S:FBH4* seedlings had shorter hypocotyls, similar to the ethylene triple response (Supplementary Fig S4). Ethylene production and *ACS7* expression were higher in plants overexpressing *FBH4*, especially until 16 h after the onset of light. In contrast, ethylene production was lower in the *fbh* mutant and *ACS7* expression decreased at night (Fig. 7). Etiolated *fbh* mutant seedlings showed longer hypocotyls compared with wild-type plants (Supplementary Fig. S4), probably due to lower ACC production in the absence of the *ACS7* activator. These results suggest that FBH4 contributes to ethylene production by inducing *ACS7* expression in the afternoon and at night. The results also indicate that a similar mechanism may exist in maturing internodes of sugarcane in which ScFBHs regulate the expression of *ScACS2*.

Our results demonstrated that the overexpression of *FBH4* in Arabidopsis caused a reduction of root length in LD-grown plants, a condition that was reverted by treatment with AgNO_3_, while the opposite effect was observed in plants overexpressing *FBH1* (Fig. 6B). Nagel *et al.* (2014) have pointed out that FBH1 seems to be a dual-function transcription factor, which acts as both an activator of *CO* (Ito *et al.*, 2012) and a repressor of *CCA1* expression (Nagel *et al.*, 2014). Our data show that FBH1 overexpression induces *ACS7* expression during the subjective day, but may inhibit *ACS7* and *ACS8* at night, while FBH4 overexpression induces *ACS8* at night (Supplementary Fig. S5D). Recently, Tian *et al.* (2015) demonstrated that overexpression of AtbHLH129, another member of subfamily 16, induces increased root length on LD-grown plants, possibly implicating other members of the subfamily, such as FBH1, in root development. This may explain why we observed differences in root phenotypes and ethylene production between *FBH1* and *FBH4* overexpressors. We highlight that all three ScFBHs are phylogenetically closer to Arabidopsis FBH4 than FBH1 (Fig. 2A). Such complex mechanisms can also explain why the roots of FBH overexpressors seem to be insensitive to ACC treatment in etiolated seedlings (Supplementary Fig. S4B).

Publications regarding the functional characterization of sugarcane transcription factors and their mechanistic regulation are still scarce, probably due to the complexity of the sugarcane genome and the instability of transgene expression (Arruda, 2012). Therefore, the use of model plants, such as Arabidopsis and tobacco, for functional studies of sugarcane genes or related genes is a common approach in sugarcane reverse genetics (Trujillo *et al.*, 2008; Begcy *et al.*, 2012; Cesarino *et al.*, 2013). Our findings support the notion that the regulation of *ACS* may be conserved in other species in addition to sugarcane, and may utilize conserved transcriptional regulators, such as FBHs.

Here we reported that three sugarcane bHLH transcription factors, ScFBH1, ScFBH2, and ScFBH3, were isolated as transcription factors that bind to the E-boxes in the *ScACS2* promoter. Our protein–protein interaction and subcellular localization analyses showed that these transcription factors form homo- and heterodimers in the nucleus. Gene expression analysis in sugarcane revealed that *ScFBHs* and *ScACS2* are expressed differentially in leaves and immature and maturing internodes, with similar expression profiles. Arabidopsis transgenic plants overexpressing *FBH1* and *FBH4* exhibit elongated hypocotyls, which is a typical phenotype of light-grown plants exposed to ACC or ethylene. Overexpression of FBH4, the closest homolog of ScFBHs, also induced ethylene production, probably due to the induction of the *ScACS2* homolog in Arabidopsis, *ACS7*. Taken together, these results suggest that ScFBHs may have a similar function to that of Arabidopsis FBHs, and may directly regulate the transcription of *ScACS2*. Our study therefore provides a new mechanistic insight into the regulatory network of ethylene synthesis in sugarcane.

## Supplementary data

Supplementary data are available at *JXB* online.

Table S1. IDs of proteins used in the ACS phylogenetic analysis.

Table S2. List of PCR primers used in this study.

Table S3. Putative transcription factor binding sites present in conserved sequences of the *ScACS2* promoter.

Table S4. List of proteins that bind to the *ScACS2* promoter identified by yeast one-hybrid screening.

Fig. S1. Transcriptional activity of the *ScACS2* promoter assessed by particle bombardment in sugarcane tissues.

Fig. S2. Phylogenetic placement of ScACS2.

Fig. S3. Promoter conserved sequences in *ScACS2* and homologous promoters from other grasses.

Fig. S4. Hypocotyl length of *FBH* overexpression and loss-of-function Arabidopsis dark-grown seedlings.

Fig. S5. Expression of Arabidopsis *ACS2*, *4*, *6*, and *8* genes in *FBH* overexpression and loss-of-function plants.

Supplementary Figures and Tables
