# Supplementary material for: The FBH family of bHLH transcription factors controls ACC synthase expression in sugarcane
Source: J Exp Bot. 2018 Mar 3;69(10):2511–25. doi: 10.1093/jxb/ery083 (PMC5920332; doi:10.1093/jxb/ery083)
Supplement: Supplementary Figures and Tables [file ery083_suppl_supplementary_figures_and_tables.pdf]

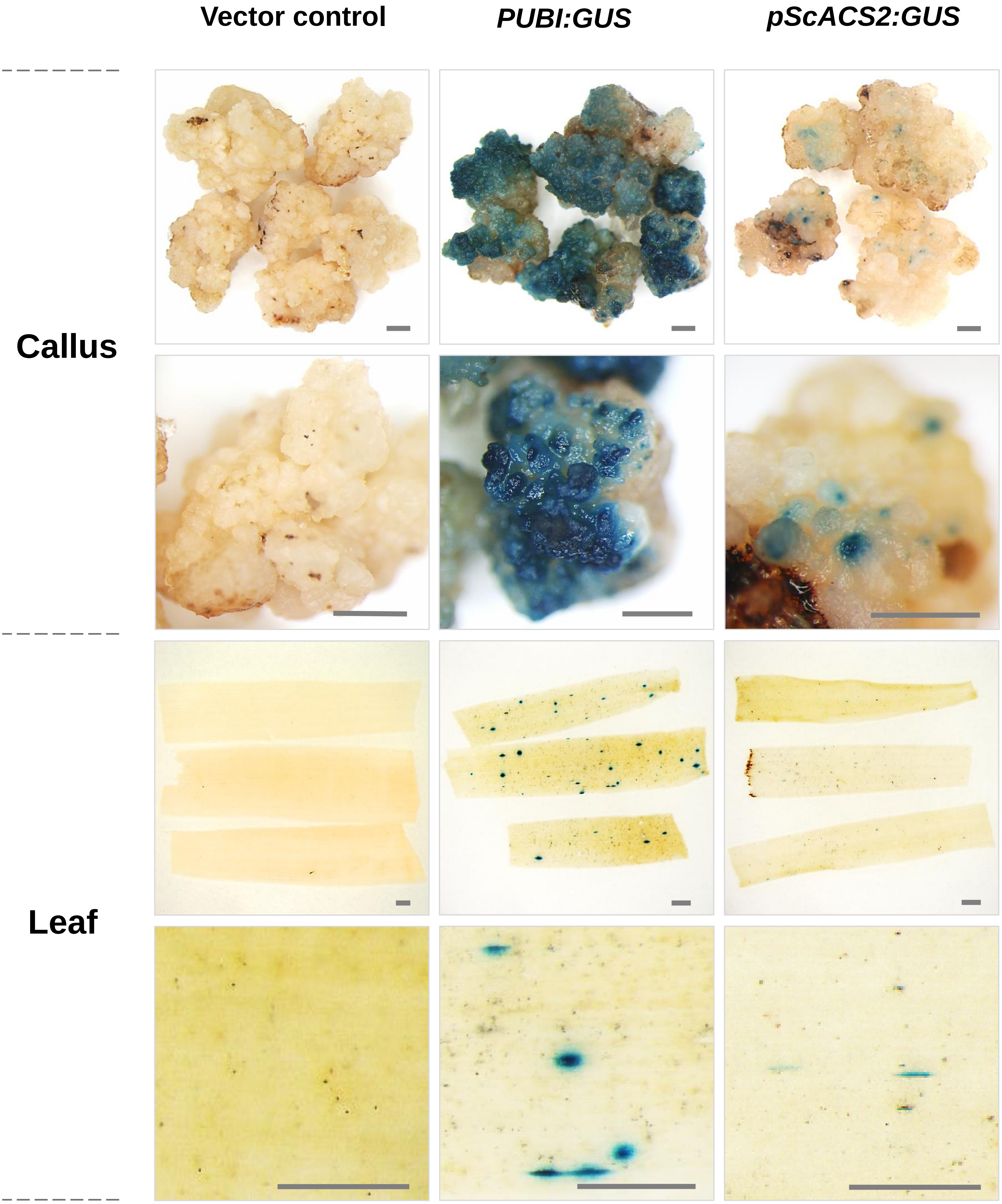


**Supplementary Fig. S1**. Confirming *ScACS2* promoter activity in sugarcane. The pHGWFS7 vector control (without promoter) and the genetic constructions *PUBI:GUS* and *PromScACS2:GUS* were transiently transformed by particle bombardment in sugarcane leaves and calli. The plant materials were incubated in the buffer containing X-Gluc for 24h and washed in 70% ethanol solution to eliminate chlorophyll from tissues. Images were taken using a DFC300FX camera coupled on a MZ10F magnifier (Leica). Bars, 1 mm.

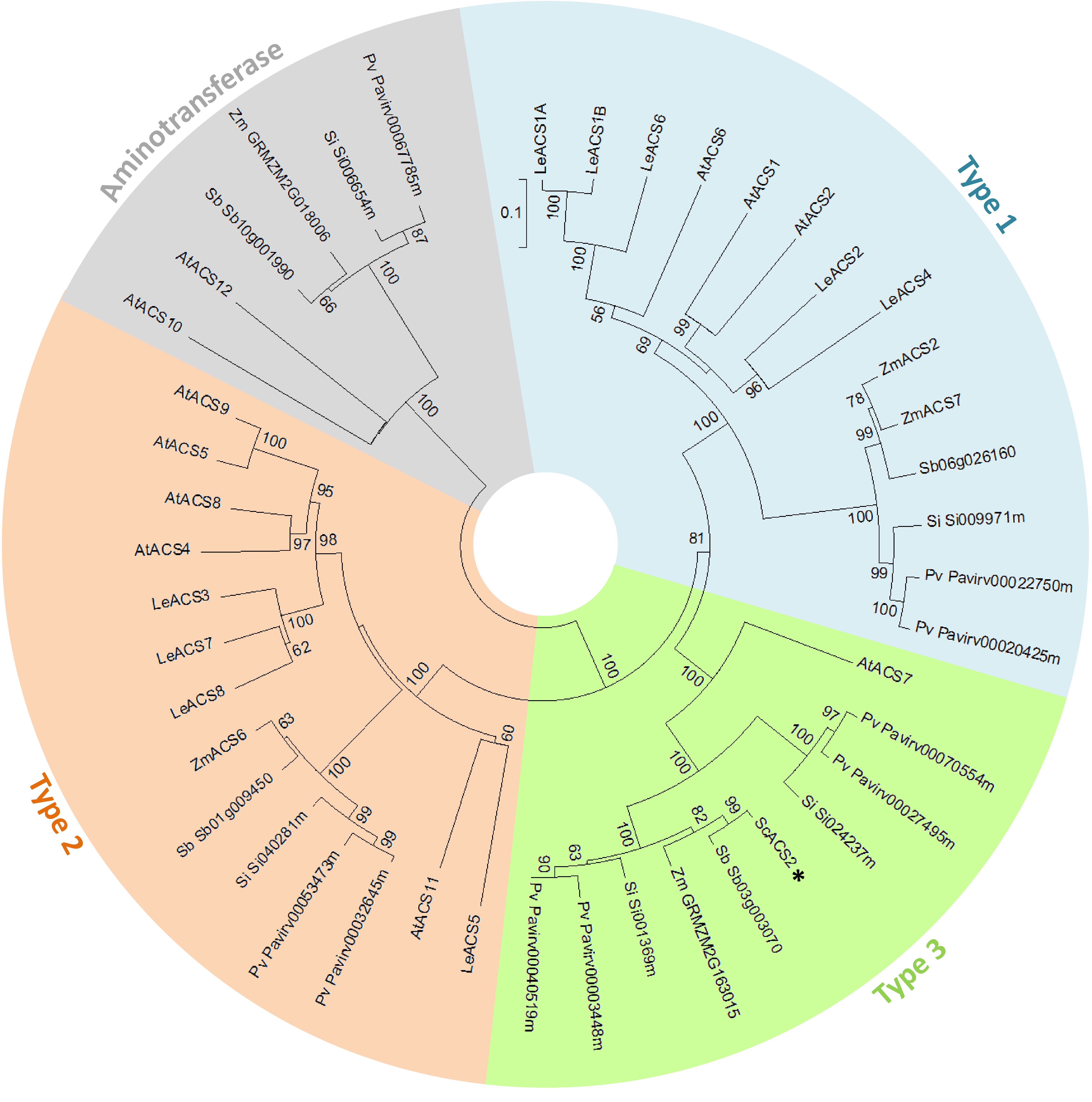


**Supplementary Fig. S2.** Phylogenetic tree of ACS proteins. The classification in type 1 (blue), 2 (orange), 3 (green) or aminotransferases (grey) is based on Arabidopsis ACS proteins (Booker and DeLong, 2015). Protein sequences were obtained from Phytozome (www.phytozome.net) or NCBI (see Supplementary Table S1). Each taxon name is represented by the ACS protein name if it is already described in the corresponding species or the Phytozome locus ID, At (*Arabidopsis thaliana*), Le (*Solanum lycopersicum*), Pv (*Panicum virgatum*), Sb (*Sorghum bicolor*), Sc (*Saccharum spp.*) Si (*Setaria italica*), Zm (*Zea mays*). The asterisk indicates the position of ScACS2. Protein sequences were aligned on WebPrank (Löytynoja and Goldman, 2010) and the Neighbor Joining tree constructed on MEGA6 (Tamura *et al.*, 2013). Bootstrap percentages of 1000 replicates are included above branches showing support values >50% for individual clades.

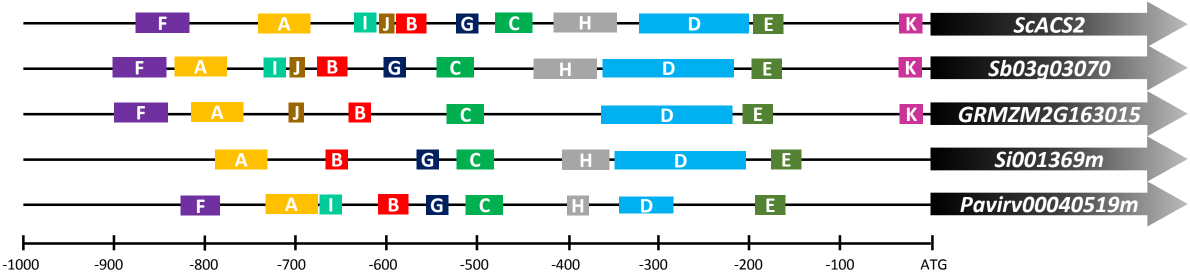


**Supplementary Fig. S3.** Promoter conserved elements on sugarcane *ScACS2*, sorghum (*Sorghum bicolor*) Sb03g03070, maize (*Zea mays*) GRMZM2G163015, foxtail millet (*Setaria italica*) Si001369m and Switchgrass (*Panicum virgatum*) Pavirv00040519m promoters. Each line represent the promoter of a different species from -1000 bp to the translational start site (ATG). Each colored box identified with a different letter (A to K) represent a conserved DNA sequence within each promoter sequence. The putative transcription factor binding sites (TFBS) identified present in these conserved regions are described in Supplementary Table S3.The given loci ID are based on Phytozome accessions.

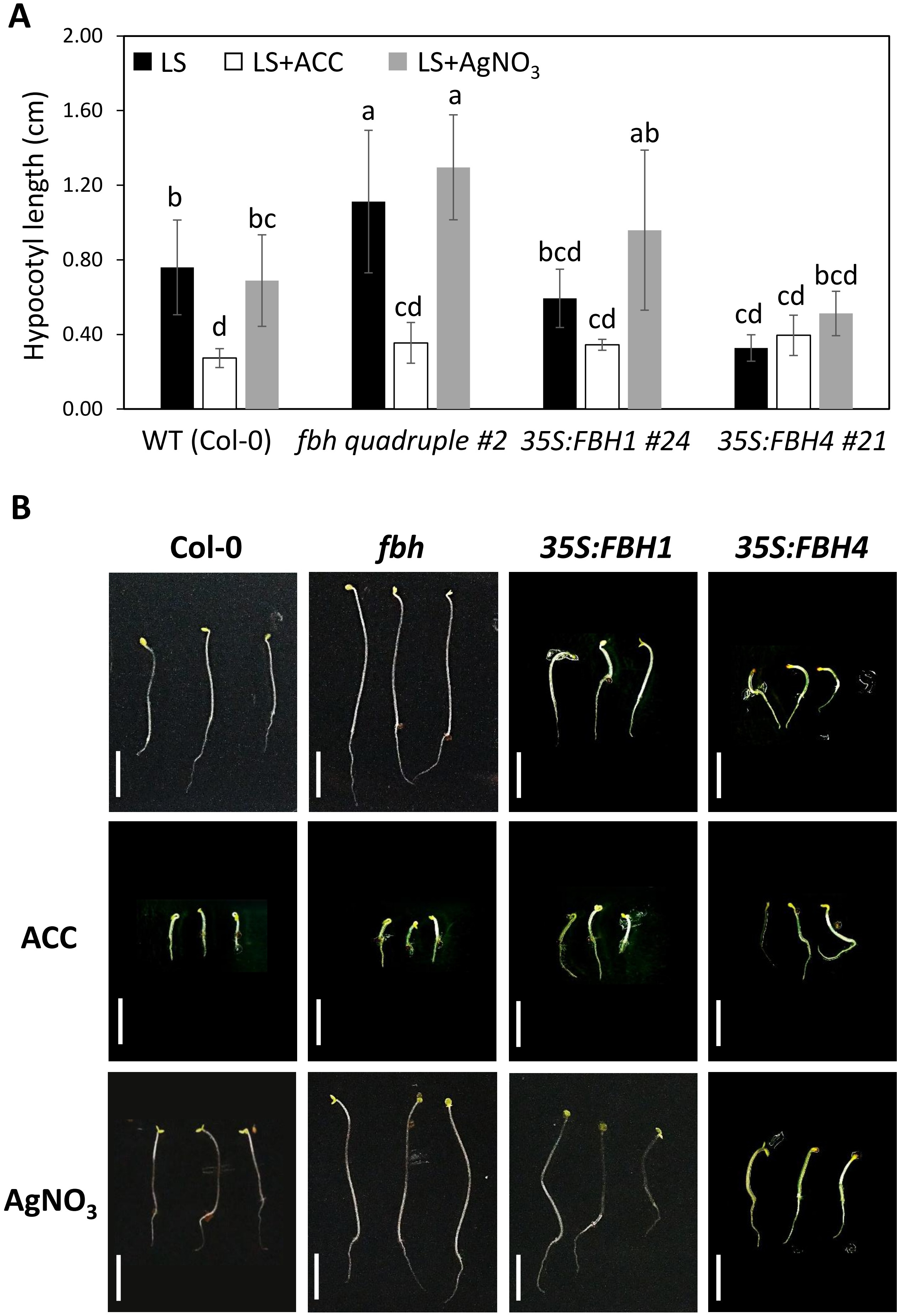


**Supplementary Fig. S4.** Effect of *FBH* overexpression and *fbh* loss-of-function in hypocotyl length in etiolated Arabidopsis seedlings. A) Hypocotyl length of Col-0, *fbh* quadruple mutant, *35S:FBH1* and *35S:FBH4* seedlings. After stratification, plants were grown on LS (Linsmaier & Skoog) media containing ACC (10 µM) or $AgNO_3$ (100 µM) for 4 days at 22°C in the dark. For each treatment, data presented are means of 3-14 seedlings ±SD. Means were compared by ANOVA and Tukey's test ($P<0.05$), the same letters above each bar indicate that there is no statistical difference between treatments. B) Hypocotyl phenotypes of 4-day-old seedlings grown with and without ACC or $AgNO_3$. Bars, 0.5 cm.

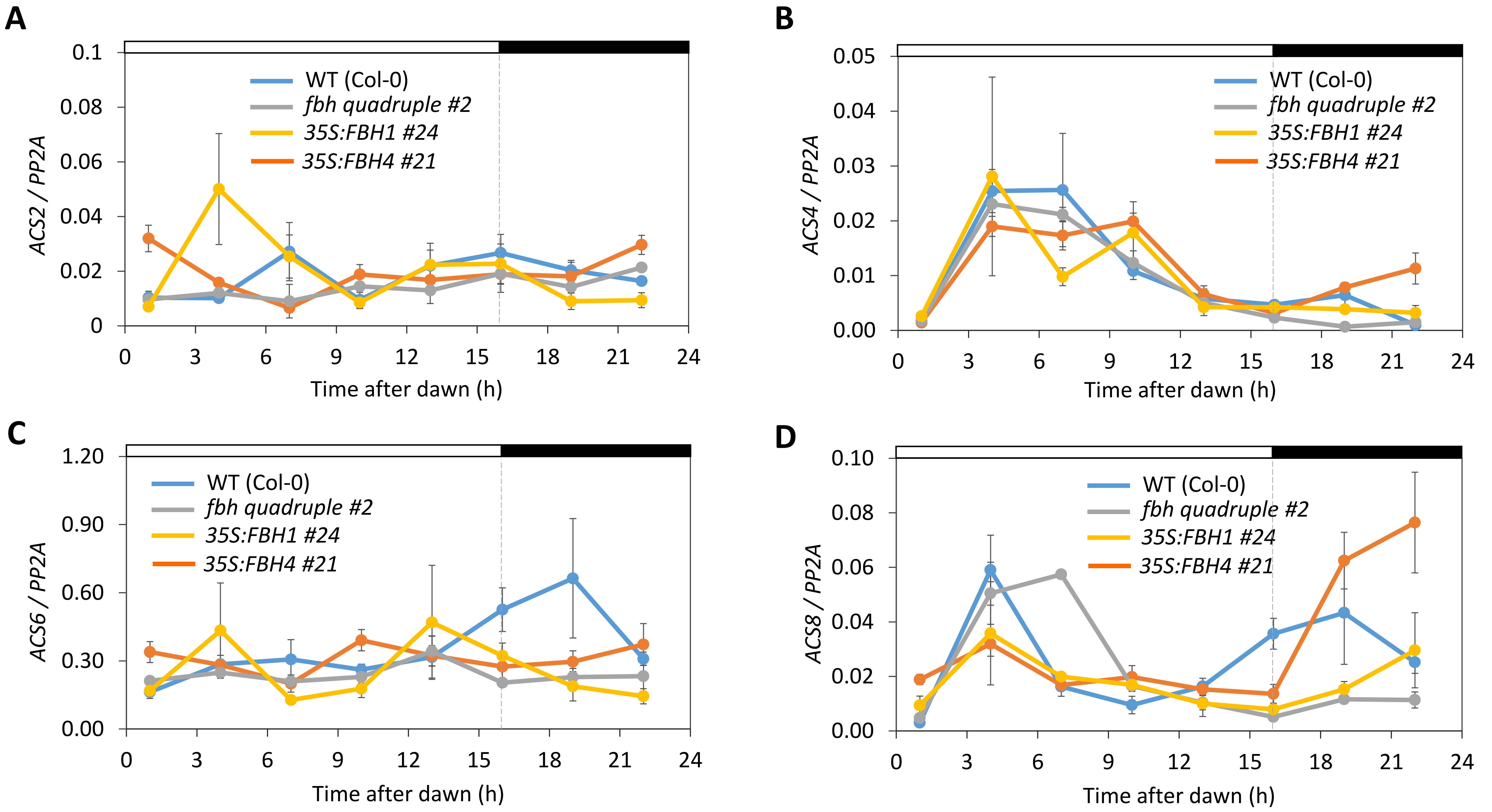


**Supplementary Fig. S5.** Effects of *FBH* overexpression and *fbh* loss-of-function in *ACS2*, *4*, *6* and *8* expression in Arabidopsis. A) Daily expression profiles of *ACS2* B) *ACS4* C) *ACS6* and D) *ACS8* in wild-type (Col-0), *fbh* quadruple mutant, *35S:FBH1*, and *35S:FBH4* plants in LD. The bars above the graphs represent light conditions: white bars, light periods; black bars, dark periods. *PP2A* gene (Hong *et al.*, 2010) was used to normalize data, and expression values were calculated by ΔCt method (Livak and Schmittgen, 2001). Values represent means ±SE from three biological replicates.

**Supplementary Table S1.** ACS proteins accession IDs in Phytozome or GenBank used in phylogenetic analysis.

| Species | ACS Name | Accession |
|---|---|---|
| *Arabidopsis thaliana* | ACS1 | AT3G61510 |
| | ACS2 | AT1G01480 |
| | ACS4 | AT2G22810 |
| | ACS5 | AT5G65800 |
| | ACS6 | AT4G11280 |
| | ACS7 | AT4G26200 |
| | ACS8 | AT4G37770 |
| | ACS9 | AT3G49700 |
| | ACS10 | AT1G62960 |
| | ACS11 | AT4G08040 |
| | ACS12 | AT5G51690 |
| *Panicum virgatum* | | Pavirv00003448m |
| | | Pavirv00070554m |
| | | Pavirv00022750m |
| | | Pavirv00040519m |
| | | Pavirv00020425m |
| | | Pavirv00032645m |
| | | Pavirv00067785m |
| | | Pavirv00027495m |
| | | Pavirv00053473m |
| *Saccharum spp.* | ScACS2 | ADZ96244.1 |
| *Setaria italica* | | Si001369m |
| | | Si009971m |
| | | Si024237m |
| | | Si006654m |
| | | Si040281m |
| *Solanum lycopersicum* | LeACS1A | AAF97614 |
| | LeACS1B | AAF97615 |
| | LeACS2 | P18485 |
| | LeACS3 | Q42881 |
| | LeACS4 | P29535 |
| | LeACS5 | AAK72430 |
| | LeACS6 | AAK72433 |
| | LeACS7 | AAC32317 |
| | LeACS8 | AAK72431 |
| *Sorghum bicolor* | | Sb01g009450 |
| | | Sb03g003070 |
| | | Sb06g026160 |
| | | Sb10g001990 |
| *Zea mays* | ZmACS2 | GRMZM2G164405 |
| | ZmACS6 | GRMZM2G054361 |
| | ZmACS7 | GRMZM5G894619 |
| | | GRMZM2G163015 |
| | | GRMZM2G018006 |

**Supplementary Table S2.** Information of primers used in this study.

| Gene | Primer name | Sequence 5'-3' |
|---|---|---|
| | **ScACS2 promoter isolation** | |
| ScACS2 | pACS2F | TCGGTGAGCAGAGACCATCACAG |
| promoter | GSP_ACS2 | GAGGAGGTCGAAGGACACCTGGTTCTC |
| | **Entry clones generation** | |
| ScACS2 | pACS2GW_F | CACCAAGCTTCACTGTCACACATGCAGCACTCC |
| promoter | pACS2GW_R | GGTACCGGCCCTTCTGCGCTTGGCTCA |
| ScFBH1 | ScFBH1_NdeI_F | CACCCATATGAATCTTAATAGACTTACTCC |
| | ScFBH1_EcoRI_R | GAATTCTTAACAAGACTTATTACCAAGAC |
| ScFBH2 | ScFBH2_NdeI_F | CACCCATATGACGTCGTCCGGGCT |
| | ScFBH2_EcoRI_R | GAATTCAGCCGGAGAACTGCTGGTGC |
| ScFBH3 | ScFBH3GW_NdeI_F | GGGGACAAGTTTGTACAAAAAAGCAGGCTCTCATA TGTATGGTGCTCCTGTTCC |
| | ScFBH3GW_EcoRI_R | GGGGACCACTTTGTACAAGAAAGCTGGGTCGAATT CAGAAGAAAATTGATCAAGCTTAC |
| | **EMSA** | |
| ScFBH2 | ScFBH2_BamHI_F | GATCGGATCCATGACGTCGTCCGGGCT |
| | ScFBH2_XhoI_R | GATCCTCGAGGCCGGAGAACTGCTGGT |
| | **qPCR analysis** | |
| ScACS2 | qPCR_ScACS2_F | ATCGCCAAGAACAAAACCAC |
| | qPCR_ScACS2_R | AAAATGGCGCCAAAAGATTA |
| ScFBH1 | qPCR_ScFBH1_F2 | ACGGCGTCAAGACTAGGAGATG |
| | qPCR_ScFBH1_R2 | CAGCAACAATCCCAATTGCGTTG |
| ScFBH2 | qPCR_ScFBH2F | CAAGACCTGCTGGAGACCTGAG |
| | qPCR_ScFBH2R | GCAGCGAACGGAGAGAAGAAG |
| ScFBH3 | qPCR_ScFBH3F | GCTCCCTTCTCCTCGTTTGG |
| | pPCR_ScFBH3R | GCCTGCTCTGCCTGCTAATG |
| ScGAPDH* | GAPDHF | CACGGCCACTGGAAGCA |
| | GAPDHR | TCCTCAGGGTTCCTGATGCC |
| AtACS2** | ACS2F | GGATGGTTTAGGATTTGCTTTG |
| | ACS2R | GCACTCTTGTTCTGGATTACCTG |
| AtACS4** | ACS4F | AACAACCTTGTGCTCACTGCT |
| | ACS4R | AGATCCCTATCAAACCCTGGA |
| AtACS6** | ACS6F | GTTCCAACCCCTTATTATCC |
| | ACS6R | CCGTAATCTTGAACCCATTA |
| AtACS7** | ACS7F | ACGGTACGATACCATTGTGGA |
| | ACS7R | GCTCGCCGTCTTTAGTTTTCT |
| AtACS8** | ACS8F | CCTTCCTTCCTTCAAGAATGC |
| | ACS8R | GAGAGTCTCGTTAGCCGGAGT |
| AtPP2AA3*** | PP2AA3F | GCGGTTGTGGAGAACATGATACG |
| | PP2AA3R | GAACCAAACACAATTCGTTGCTG |

* Iskandar et al. (2004), ** Li et al. (2012), *** Hong et al. (2010)

**Supplementary Table S3.** Transcription factors binding sites (TFBS) prediction on *ScACS2* gene promoter. Promoter conserved elements (PCE, see Supplementary Fig. S2) were identified by alignments with homologous promoters from sorghum (Sb03g03070), maize (GRMZM2G163015), foxtail millet (Si001369m) and switchgrass (Pavirv00040519m) and putative TFBS were identified within these regions using the PlantPan 2 database (Chow *et al.*, 2016). Only DNA motifs with scores higher than 0.9 were considered. The TFBS DNA motifs were manually checked on the references provided.

| PCE | TFBS | Strand | Score | Sequence | Related TF | Associated function | Reference |
|---|---|---|---|---|---|---|---|
| A | AT-HOOK | + | 1.00 | caAATATtcc | AT-HOOK | Regulates chromatin dynamics | (Weirauch *et al.*, 2014) |
| | GATA | - | 1.00 | CATG | GATA/ZIM/TIFY | Light responsiveness | (Weirauch *et al.*, 2014) |
| | NAC | - | 1.00 | ACGGCatc | NAC042/JUB1 | Leaf senescence | (Wu *et al.*, 2012) |
| | MYB | + | 1.00 | AACGG | HYP | Regulates CycB1 gene in Arabidopsis | (Planchais *et al.*, 2002) |
| | SEF3 | + | 1.00 | AACCCa | GmSEF3 | Regulates β-conglicinin during seed development | (Allen *et al.*, 1989) |
| | MYB | + | 1.00 | cAACGG | MYB2 | Dehydration response | (Abe *et al.*, 2003) |
| | MYB/GARP/G2 | + | 0.98 | caaaTATTCc | KAN1, ARR, G2-like | Regulates lateral organ polarity | (Franco-Zorrilla *et al.*, 2014) |
| | E2F | + | 0.94 | aTTCCCct | E2F | Cell cycle progression | (Vandepoele *et al.*, 2005) |
| B | GATA | - | 1.00 | CATCT | GATA/ZIM/TIFY | Light responsiveness | (Weirauch *et al.*, 2014) |
| | E-box | + | 1.00 | CATCTg | bHLH | Plant development and stress responses | (Franco-Zorrilla *et al.*, 2014) |
| | BES1/BZR1 | - | 1.00 | cGCACG | BZR1 | Brassinosteroid-response element | (He *et al.*, 2005) |
| | RHE | + | 1.00 | TGnnnGCACGa | - | Regulate EXPA7 expression in roots | (Kim *et al.*, 2006) |
| | SITE II element | - | 0.95 | cGCCCA | - | Cytc-1 and Cytc-2 genes regulation | (Welchen and Gonzalez, 2005) |
| C | E-box | + | 1.00 | CAGCTg | bHLH | Plant development and stress responses | (Franco-Zorrilla *et al.*, 2014) |
| | P1BS | + | 1.00 | GCATAttc | PHR-1 | Phosphatestarvation response | (Rubio *et al.*, 2001) |
| | MYB/GARP/G2 | + | 0.98 | caaaTATTCc | KAN1, ARR, G2-like | Lateral organ polarity determination | (Franco-Zorrilla *et al.*, 2014) |
| | C2H2 | + | 0.93 | aaCAGCTgga | ZF-C2H2 | Pant development and stress responses | (Franco-Zorrilla *et al.*, 2014) |
| D | DOF | + | 1.00 | AAAGC | ZF-DOF | Plant development and stress responses | (Yanagisawa and Schmidt, 1999) |
| | GATA | + | 1.00 | TGATT | GATA/ZIM/TIFY | Light responsiveness | (Weirauch *et al.*, 2014) |
| | ZF-HD | + | 1.00 | ATTAT | AtHB33 | Floral development | (Tan and Irish, 2006) |
| | ARR1 | + | 1.00 | TGATT | ARR1/ARR2 | Cytokinin and dehydration response | (Sakai *et al.*, 2000) |
| | Homeodomain | + | 1.00 | ttTGACAttt | KNAT6/BUM1/STM | Shoot apical meristem development | (Weirauch *et al.*, 2014) |
| | BIHD1OS | - | 1.00 | TGACA | OsBIH1 | Biotic stress response regulation | (Luo *et al.*, 2005) |

| | | | | | | | |
|---|---|---|---|---|---|---|---|
| | NOD | + | 1.00 | aTCTTT | - | Root nodules formation regulation | (Stougaard *et al.*, 1990) |
| | AT-HOOK | - | 0.99 | ATTATatcg | AT-HOOK | Regulates chromatin dyamics | (Weirauch *et al.*, 2014) |
| | MYB | - | 1.00 | TGGTTt | MYB1 | Dehydration responsiveness | (Abe *et al.*, 2003) |
| | DOF | - | 0.99 | acaTCTTTtg | ZF-DOF | Plant development and stress responses | (Yanagisawa and Schmidt, 1999) |
| | MYB | - | 0.97 | tggTGGTTtc | MYB46 | Secondary wall biosynthesis regulation | (Franco-Zorrilla *et al.*, 2014) |
| | W-box | + | 0.95 | TTGACa | WRKY | Biotic stress response regulation | (Weirauch *et al.*, 2014) |
| F | NOD | - | 1.00 | AAGAG | - | Root nodules formation regulation | (Stougaard *et al.*, 1990) |
| | E2F | + | 1.00 | aTTCCCgg | E2F | Regulates cell cycle progression | (Vandepoele *et al.*, 2005) |
| | C2H2 | - | 0.94 | ccgAGCTGtg | ZF-C2H2 | Plant development and stress responses | (Franco-Zorrilla *et al.*, 2014) |
| G | DOF | + | 1.00 | AAAGG | ZF-DOF | Plant development and stress responses | (Weirauch *et al.*, 2014) |
| | ARF | + | 0.96 | ggCGACAgc | ARF11/ARF19 | Auxin and ethylene induced responses | (Weirauch *et al.*, 2014) |
| | E2F | - | 0.94 | ctGCGAAa | E2F | Cell cycle progression | (Vandepoele *et al.*, 2005) |
| H | GATA | - | 1.00 | AATCA | GATA/ZIM/TIFY | Involved in light responsiveness | (Weirauch *et al.*, 2014) |
| | ARR1 | - | 1.00 | AATCA | ARR1/ARR2 | Cytokinin and dehydration response | (Sakai *et al.*, 2000) |
| | ATHB2 | + | 1.00 | CAATCattg | HD-ZIP/AtHB2 | Regulates cell expansion and prolifferation | (Sessa *et al.*, 1993) |
| | WOX | - | 0.99 | tcAATCAt | WOX14 | Stem cells maintanance | (Weirauch *et al.*, 2014) |
| I | W-box | + | 1.00 | TGACC | OsWRKY71 | Aleurone development | (Zhang *et al.*, 2004) |
| | SORLIP2 | + | 1.00 | GGGCC | - | Present in light regulated genes | (Hudson and Quail, 2003) |
| | Q-Element | - | 1.00 | tGACCT | - | Present in polen expressed genes promoters | (Hamilton *et al.*, 1998) |
| J | AT-HOOK | + | 1.00 | gattAATAAa | AT-HOOK | Regulates chromatin dyamics | (Weirauch *et al.*, 2014) |
| | GATA | + | 1.00 | GGATT | GATA/ZIM/TIFY | Light responsiveness | (Weirauch *et al.*, 2014) |
| | ZF-HD | + | 1.00 | ATTAA | AtHB33 | Involved in floral development | (Tan and Irish, 2006) |
| | MYB/ARR | + | 1.00 | GGATT | ARR14 | Member of response regulator type-B | (Weirauch *et al.*, 2014) |
| | WOX | - | 1.00 | ggATTAA | WUS1 | Embryogenesis, oogenesis and flowering | (Weirauch *et al.*, 2014) |
| | ANAERO1 | + | 1.00 | AAACAaa | - | Anaerobic responsive elemento | (Mohanty *et al.*, 2005) |
| | AACA Core | + | 1.00 | AACAAac | - | Related to endosperm specific expression | (Wu *et al.*, 2000) |
| | Homeodomain | + | 0.95 | ggaTTAATaa | AtHB12 | Dehydration responsiveness | (Weirauch *et al.*, 2014) |

**References of Supplementary Table S3**


**Abe H, Urao T, Ito T, Seki M, Shinozaki K, Yamaguchi-Shinozaki K**. 2003. Arabidopsis AtMYC2 (bHLH) and AtMYB2 (MYB) function as transcriptional activators in abscisic acid signaling. The Plant Cell **15**, 63–78.

**Allen RD, Bernier F, Lessard PA, Beachy RN**. 1989. Nuclear factors interact with a soybean beta-conglycinin enhancer. The Plant Cell **1**, 623–631.

**Chow C-N, Zheng H-Q, Wu N-Y, Chien C-H, Huang H-D, Lee T-Y, Chiang-Hsieh Y-F, Hou P-F, Yang T-Y, Chang W-C**. 2016. PlantPAN 2.0: an update of plant promoter analysis navigator for reconstructing transcriptional regulatory networks in plants. Nucleic Acids Research **44**, D1154–D1160.

**Franco-Zorrilla JM, López-Vidriero I, Carrasco JL, Godoy M, Vera P, Solano R**. 2014. DNA-binding specificities of plant transcription factors and their potential to define target genes. Proceedings of the National Academy of Sciences of the United States of America **111**, 2367–2372.

**Hamilton DA, Schwarz YH, Mascarenhas JP**. 1998. A monocot pollen-specific promoter contains separable pollen-specific and quantitative elements. Plant Molecular Biology **38**, 663–669.

**He J, Gendrom J, Sun Y, Gampala S, Gendron N, Sun C, Wang Z**. 2005. BZR1 is a transcriptional repressor with dual roles in brassinosteroid homeostasis and growth responses. Science **307**, 1634–1638.

**Hudson ME, Quail PH**. 2003. Identification of Promoter Motifs Involved in the Network of Phytochrome A-Regulated Gene Expression by Combined Analysis of Genomic Sequence and Microarray Data. Plant Physiology **133**, 1605–1616.

**Kim DW, Lee SH, Choi S-B, Won S-K, Heo Y-K, Cho M, Park Y-I, Cho H-T**. 2006. Functional Conservation of a Root Hair Cell-Specific cis-Element in Angiosperms with Different Root Hair Distribution Patterns. The Plant Cell **18**, 2958–2970.

**Luo H, Song F, Goodman RM, Zheng Z**. 2005. Up-regulation of OsBIHD1, a rice gene encoding BELL homeodomain transcriptional factor, in disease resistance responses. Plant Biology **7**, 459–468.

**Mohanty B, Krishan S, Swarup S, Bajic VB**. 2005. Detection and preliminary analysis of motifs in promoters of anaerobically induced genes of different plant species. Annals of Botany **96**, 669–681.

**Planchais S, Perennes C, Glab N, Mironov V, Inze D, Bergounioux C**. 2002. Characterization of cis-acting element involved in cell cycle phase-independent activation of Arath;CycB1;1 transcription and identification of putative regulatory proteins. Plant Molecular Biology **50**, 111–127.

**Rubio V, Linhares F, Solano R, Martin AC, Iglesias J, Leyva A, Paz-Ares J**. 2001. A conserved MYB transcription factor involved in phosphate starvation signaling both in vascular plants and in unicellular algae. Genes & Development **15**, 2122–2133.

**Sakai H, Aoyama T, Oka A**. 2000. Arabidopsis ARR1 and ARR2 response regulators operate as transcriptional activators. The Plant Journal **24**, 703–711.

**Sessa G, Morelli G, Ruberti I**. 1993. The Athb-1 and -2 HD-Zip domains homodimerize forming complexes of different DNA binding specificities. The EMBO Journal **12**, 3507–3517.

**Stougaard J, Jorgensen JE, Christensen T, Kuhle A, Marcker KA**. 1990. Interdependence and nodule specificity of cis-acting regulatory elements in the soybean leghemoglobin lbc3 and N23 gene promoters. Molecular & General Genetics **220**, 353–360.

**Tan QK-G, Irish VF**. 2006. The Arabidopsis Zinc Finger-Homeodomain genes encode proteins with unique biochemical properties that are coordinately expressed during floral development. Plant Physiology **140**, 1095–1108.

**Vandepoele K, Vlieghe K, Florquin K, Hennig L, Beemster GTS, Gruissem W, Van de Peer Y, Inze D, De Veylder L**. 2005. Genome-wide identification of potential plant E2F target genes. Plant Physiology **139**, 316–328.

**Weirauch MT, Yang A, Albu M, *et al.*** 2014. Determination and inference of eukaryotic transcription factor sequence specificity. Cell **158**, 1431–1443.

**Welchen E, Gonzalez DH**. 2005. Differential expression of the Arabidopsis cytochrome c genes Cytc-1 and Cytc-2. Evidence for the involvement of TCP-domain protein-binding elements in anther- and meristem-specific expression of the Cytc-1 gene. Plant Physiology **139**, 88–100.

**Wu A, Allu AD, Garapati P, *et al.*** 2012. JUNGBRUNNEN1, a reactive oxygen species–responsive NAC transcription factor, regulates longevity in Arabidopsis. The Plant Cell **24**, 482–506.

**Wu C, Washida H, Onodera Y, Harada K, Takaiwa F**. 2000. Quantitative nature of the Prolamin-box, ACGT and AACA motifs in a rice glutelin gene promoter: minimal cis-element requirements for endosperm-specific gene expression. The Plant Journal **23**, 415–421.

**Yanagisawa S, Schmidt RJ**. 1999. Diversity and similarity among recognition sequences of Dof transcription factors. The Plant Journal **17**, 209–214.

**Zhang Z-L, Xie Z, Zou X, Casaretto J, Ho T-HD, Shen QJ**. 2004. A rice WRKY gene encodes a transcriptional repressor of the gibberellin signaling pathway in aleurone cells. Plant Physiology **134**, 1500–1513.

**Supplementary Table S4.** NCBI Blast based functional annotation of clones identified in yeast one-hybrid. Sugarcane leaf (L) and culm (C) cDNA libraries were screened using the *ScACS2* promoter as a bait sequence. The loci IDs of the best BlastX hit against sorghum and Arabidopsis genomes available at Phytozome are provided.

| # | Clones | Putative function | Library | Sorghum best hit | Arabidopsis best hit |
|---|---|---|---|---|---|
| 1 | 2 | Basic Helix-loop-Helix transcription factor | L | Sb03g042860 | At2g42280 (FBH4) |
| 2 | 5 | Basic Helix-loop-Helix transcription factor | L,C | Sb07g027810 | At2g42280 (FBH4) |
| 3 | 2 | Basic Helix-loop-Helix transcription factor | L | Sb02g028300 | At2g42280 (FBH4) |
| 4 | 1 | Golden2-like transcription factor (GLK) | L | Sb10g008400 | At5g44190 (GLK2) |
| 5 | 1 | Aluminium induced protein | L | Sb08g002850 | At5g19140 (AILP1) |
| 6 | 1 | Auxin responsive fator (ARF) | C | Sb04g022830 | No hit |
| 7 | 1 | Coronatine insensitive protein 1 (COI1) | C | Sb03g040150 | At2g39940 (COI1) |
| 8 | 1 | Lecithin-cholesterol acyltransferase-like | L | Sb03g002740 | At1g27480 |
| 9 | 1 | Vacuolar cátion/próton exchanger | C | Sb03g024820 | At3g51860 (CAX3) |
| 10 | 1 | Unknown protein | L | Sb07g000210 | At1g10820 |
| 11 | 1 | Unknown protein | L | Sb10g003740 | At1g53450 |

AILP: Aluminum induced protein; ARF: Auxin responsive factor; CAX: Calcium exchanger; COI: Coronatine insensitive; FBH: Flowering bHLH; GLK: Golden2-like.
